# Nanoindentation Response of Structural Self-Healing Epoxy Resin: A Hybrid Experimental–Simulation Approach

**DOI:** 10.3390/polym16131849

**Published:** 2024-06-28

**Authors:** Giovanni Spinelli, Rosella Guarini, Evgeni Ivanov, Elisa Calabrese, Marialuigia Raimondo, Raffaele Longo, Liberata Guadagno, Luigi Vertuccio

**Affiliations:** 1 Faculty of Transport Sciences and Technologies, University of Study “Giustino Fortunato”, Via Raffaele Delcogliano 12, 82100 Benevento, Italy; 2 Open Laboratory on Experimental Micro and Nano Mechanics, Institute of Mechanics, Bulgarian Academy of Sciences, Acad. G. Bonchev Str., Block 4, 1113 Sofia, Bulgaria; rgrosagi@gmail.com (R.G.); ivanov_evgeni@yahoo.com (E.I.); 3 Department of Industrial Engineering, University of Salerno, Via Giovanni Paolo II, 84084 Fisciano, Italy; elicalabrese@unisa.it (E.C.); mraimondo@unisa.it (M.R.); rlongo@unisa.it (R.L.); 4 Department of Engineering, University of Campania “Luigi Vanvitelli”, Via Roma 29, 81031 Aversa, Italy; luigi.vertuccio@unicampania.it

**Keywords:** nanoindentation, epoxy resin, mechanical properties, self-healing

## Abstract

In recent years, self-healing polymers have emerged as a topic of considerable interest owing to their capability to partially restore material properties and thereby extend the product’s lifespan. The main purpose of this study is to investigate the nanoindentation response in terms of hardness, reduced modulus, contact depth, and coefficient of friction of a self-healing resin developed for use in aeronautical and aerospace contexts. To achieve this, the bifunctional epoxy precursor underwent tailored functionalization to improve its toughness, facilitating effective compatibilization with a rubber phase dispersed within the host epoxy resin. This approach aimed to highlight the significant impact of the quantity and distribution of rubber domains within the resin on enhancing its mechanical properties. The main results are that pure resin (EP sample) exhibits a higher hardness (about 36.7% more) and reduced modulus (about 7% more), consequently leading to a lower contact depth and coefficient of friction (11.4% less) compared to other formulations that, conversely, are well-suited for preserving damage from mechanical stresses due to their capabilities in absorbing mechanical energy. Furthermore, finite element method (FEM) simulations of the nanoindentation process were conducted. The numerical results were meticulously compared with experimental data, demonstrating good agreement. The simulation study confirms that the EP sample with higher hardness and reduced modulus shows less penetration depth under the same applied load with respect to the other analyzed samples. Values of 877 nm (close to the experimental result of 876.1 nm) and 1010 nm (close to the experimental result of 1008.8 nm) were calculated for EP and the toughened self-healing sample (EP-R-160-T), respectively. The numerical results of the hardness provide a value of 0.42 GPa and 0.32 GPa for EP and EP-R-160-T, respectively, which match the experimental data of 0.41 GPa and 0.30 GPa. This validation of the FEM model underscores its efficacy in predicting the mechanical behavior of nanocomposite materials under nanoindentation. The proposed investigation aims to contribute knowledge and optimization tips about self-healing resins.

## 1. Introduction

In recent years, polymers with different functional properties have increased the interest of the industrial and academic community [1,2,3,4,5,6]. In particular, self-healing polymers have garnered significant attention due to their ability to partially or totally restore material properties, thereby extending product lifespans. Additional advantages include minimizing the environmental footprint and reducing resource depletion [7,8,9,10].

Previous research on self-healing polymeric materials has yielded numerous healing mechanisms, with current efforts increasingly directed toward developing advanced systems capable of supporting multiple healing cycles [11,12,13]. Thermosetting polymers, especially epoxies, are extensively used in coatings, adhesives, and structural components due to their high mechanical strength, thermal stability, and resistance to chemicals and corrosion [14,15]. However, their highly crosslinked network limits intrinsic healing mechanisms [16]. Modifications to epoxies aim to enhance healing functionality, with various strategies reported in the literature considering that self-healing polymers can be classified as either extrinsic or intrinsic [17,18,19,20,21,22].

Compared to extrinsic capsule-based and vascular self-healing materials, intrinsic self-healing materials are simpler and alleviate challenges associated with integrating healing agents, ensuring compatibility, and maintaining catalyst stability [23]. Nevertheless, many intrinsic self-healing systems lack mechanical and chemical properties suitable for structural applications. Hence, epoxies, renowned for their suitability in such applications, are modified to enhance mechanical properties combined with efficient healing capabilities. Different studies in the literature are focused on these objectives. Outwater et al. proposed a novel method to measure the fracture energy of cast epoxy resin in the presence of different agents [24]. In this pioneering work, it is observed that upon heating above the glass transition temperature (Tg), the crack spontaneously healed, resulting in a fresh joint with a fracture energy comparable to that of the original material. The authors attributed this phenomenon to micro-Brownian motions facilitating the local flow and ensuring sound bond formation [24]. Likewise, Rahmathullah et al. implemented a healing protocol for addressing an arrested crack within fully cured epoxy–amine thermosets [25]. Several studies reported that the explanation for the superficial repair of epoxy cracks involves shape-memory mechanisms. Luo and Mather presented the preparation and characterization of novel shape memory-assisted self-healing (SMASH) coatings [26]. These coatings exhibit a phase-separated morphology of electrospun thermoplastic poly(ε-caprolactone) (PCL) fibers dispersed randomly within a shape-memory epoxy matrix. [26]. Nelson et al. reported a temperature-dependent recovery of atomic force microscope tip-formed indentations in a thermoset shape memory polymer, highlighting with their study that the shape recovery is more complete for higher anneal temperature [27]. Cho et al. investigated self-healing polymer coatings to study the differences in using two distinct approaches: first, the catalyst is microencapsulated in the epoxy resin, whereas the healing agent (siloxanes) was as phase-separated droplets. In the second approach, both phases were also encapsulated and dispersed in the coating matrix. As a result, it was found that this second solution appears valuable in cases where the matrix can react with the healing agent [28]. Our previous studies showed an intrinsic self-healing system comprising an epoxy resin covalently modified with a rubber phase and self-healing fillers dispersed in the epoxy system [29,30]. Incorporating the elastomer component decreases the rigidity of the epoxy chains and stimulates the activation of an auto-repair mechanism driven by hydrogen bonding interactions. Moreover, rubber-enhanced thermosetting resins demonstrate several advantages over their unmodified counterparts. In a previous paper [31], the authors examined the influence of the functionalization temperature (120 °C and 160 °C) to obtain an efficient toughened material. In particular, FT/IR and DMA analyses were used to investigate the functionalization of the epoxy precursor with the elastomeric phase at two different temperatures. The results showed a higher fraction of rubber phase bonded to the epoxy precursor at the higher functionalization temperature of 160 °C compared to 120 °C. Moreover, self-healing tests via tapered double cantilever beam (TDCB) also demonstrated a significant healing efficiency for the system functionalized at 160 ºC compared to that at 120 °C. In the present work, the nanocomposites obtained through a functionalization temperature of 160 °C were further characterized by nanoindentation tests in terms of hardness, reduced modulus, contact depth, and coefficient of friction (COF) at scratch.

Microstructural analysis and nanoindentation characterization have recently generated increasing interest in exploring mechanical properties at the nanoscale level further [32].

The nanoindentation technique is successfully presented in the literature to study the creep behavior of supercrystalline nanocomposites (SCNCs) with different levels of organic crosslinking [33].

The improvement of mechanical properties in terms of elastic modulus due to the incorporation of nanoclay platelets into epoxy resin has also been analyzed via nanoindentation [34].

The innovative method based on accelerated property mapping (XPM), which provides measuring maps of local mechanical properties (reduced modulus, hardness), is also employed in the current investigation. All observed results are compared with those related to the unmodified hosting epoxy resin adopted as a reference.

Finally, finite element simulation studies are undertaken to predict additional mechanical properties of the formulated samples, leveraging select experimental results.

This experimental/computational hybrid approach has been successfully presented in the literature to introduce and support laboratory tests concerning the nanoindentation of heterogeneous nanocomposite materials [35]. Similarly, mathematical approaches have been proposed to interpret the results of nanoindentation characterization concerning the elastic and viscoelastic properties of polymer nanocomposites [36].

This study presents an in-depth nanomechanical characterization of various composite samples, providing detailed insights into their mechanical properties regarding hardness, reduced modulus, contact depth, and coefficient of friction. Advanced XPM analysis offers a high-resolution understanding of the material properties at the microscale, revealing significant variations and trends that have not been previously reported. The research uniquely integrates experimental nanoindentation data with a simulation study based on the finite element method (FEM). This dual approach ensures a comprehensive validation of the experimental results, enhancing the reliability and accuracy of the findings. A key novelty of this work is the meticulous reproduction of the experimental setup within the finite element model, particularly concerning the indentation tip geometry. This study minimizes approximation errors by closely matching the simulation conditions to the actual experimental parameters, resulting in a good correlation between experimental and simulated results. By accurately modeling the indentation process and avoiding common approximation errors, the study sets a benchmark for future research. This detailed approach ensures that the mechanical properties derived from simulations are as close as possible to those observed experimentally, reducing discrepancies and enhancing the overall reliability of the results. The excellent agreement between the experimental nanoindentation data and the finite element simulations serves as a strong validation of the model. This successful validation highlights the potential of using such detailed simulations to predict the nanomechanical properties of new composite materials, strongly reducing experimental tests.

## 2. Materials

### 2.1. Materials: Chemical Formulations

Although the samples are part of a broader set of composites investigated differently in a previous study [31], their preparation is recalled here for clarity and completeness.

The prepared samples consist of a toughened epoxy matrix including the precursor (Gurit Holding, Wattwil, Switzerland) “3,4-Epoxycyclohexylmethyl-3′,4′-epoxycyclohexane carboxylate” (acronyms ECC and chemical formula C_14_H_20_O_4_) and the hardener agent (Gurit Holding, Wattwil, Switzerland) “Methylhexahydrophthalicanhydride” (acronyms MHHPA and chemical formula C_9_H_12_O_3_) in a 1:1 ratio.

The liquid rubber that acts as a toughening agent, Carboxyl-Terminated Butadiene Acrylonitrile Copolymer (henceforth designed as “R”), was used at a concentration of 5 wt% with respect to the overall mixture. It was supplied by Hycar reactive liquid polymers, featuring a molecular weight (Mn) of 3600 and containing terminal carboxy groups (with a COOH content of 0.67 × 10^−3^ equiv/g of CTBN and 18 *w*/*w*% of CN). The catalyst, triphenylphosphine (PPh3, Merck KGaA, Darmstadt, Germany), was added to facilitate the functionalization reaction of the epoxy precursor (ECC).

**Figure 1:**
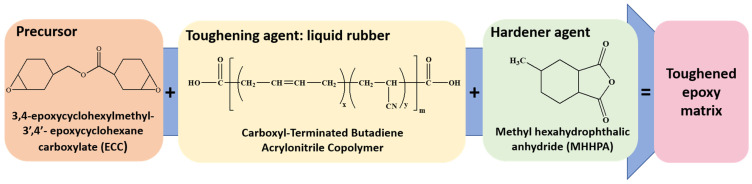
Chemical formulas of the precursor, toughening agent, and hardener agent, from left to right, respectively.

Figure 1 reports (from left to right) the structural chemical formulas of the following components: epoxy precursor (ECC), liquid rubber (R), and hardener (MHHPA), respectively, whereas Table S1 of the “Supplementary electronic materials (S.E.M.)” summarizes the quantities in grams and percentages of individual components required to produce 23.58 g of the complete mixture, here named EP-R-160.

Based on the selected composition, the initial nucleophilic attack trough triphenylphosphine unlocks 9.15 × 10^−3^ mol of oxirane rings, thus still leaving unreacted 7.0 × 10^−2^ mol of oxirane rings, which represent 88% of the rings initially present. The moles of the terminal carboxylic groups are equivalent to 0.804 × 10^−3^, which results in a quantity slightly lower than the opened oxirane rings (9.15 × 10^−3^ mol).

This decision was taken to prevent the formation of extensive elastomeric domains within the resin, as later confirmed through SEM analysis. Drawing from both previously published research [37] and the expertise of the authors, it was observed that a reduction in triphenylphosphine content (or an increase in the rubber phase relative to the amount of PPh3) leads to larger elastomer domains, resulting in significant local inhomogeneity on the scale of hundreds or thousands of microns.

**Figure 2:**
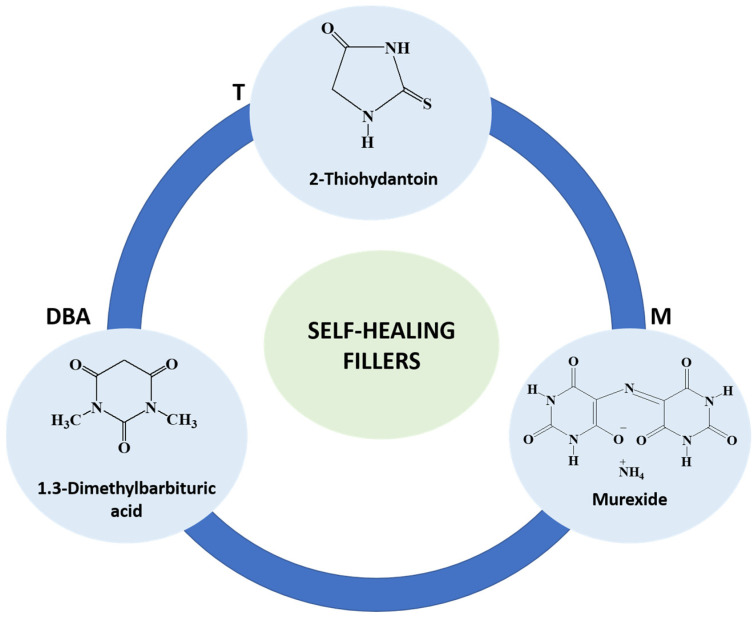
Chemical structure of molecules acting as self-healing filler.

The self-healing additives, with chemical structures depicted in Figure 2, 1.3-Dimethylbarbituric acid (DBA), 2-Thiohydantoin (T), and Murexide (M) (all sourced from Merck KGaA, Darmstadt, Germany) were included at a concentration of 0.42 wt%. The capacity of these additives to initiate self-repair mechanisms through hydrogen bonding interactions has been previously established. This was demonstrated in prior research utilizing a nano-enhanced resin composed of a tetra-functional epoxy precursor cured with 4,4-diamino diphenyl sulfone [29].

### 2.2. Formulation of Epoxy Sample

#### 2.2.1. Functionalized Epoxy Precursor

The ECC-R-160 functionalized precursor liquid blends consisted of epoxy precursor molecules chemically modified with the rubber phase. These blends were prepared by mechanically stirring a mixture of ECC epoxy precursor, elastomer R, and PPh3 catalyst for 15 h at a temperature of 160 °C.

#### 2.2.2. Functionalized Epoxy Samples

The cured epoxy samples, labeled as EP-R-160, were prepared by adding the hardener MHHPA to the functionalized precursor ECC-R-160 using magnetic stirring for 20 min at room temperature. After degassing for 2 h at room temperature, the mixture underwent polymerization in an oven through a curing cycle comprising 1 h at 80 °C, followed by 20 min at 120 °C, and then 1 h at 180 °C. During the curing process, the samples were initially placed in the oven at 80 °C. After the first hour of curing, the oven temperature was ramped up from 80 °C to 120 °C at a rate of 10 °C/min, while the actual heating rate measured with a probe was 4 °C/min. The same procedure was followed for the transition from 120 °C to 180 °C. For comparison, an EP sample, representing the cured epoxy matrix without the rubber phase, was prepared by mixing the ECC precursor and MHHPA hardener following the aforementioned procedure. Functionalized epoxy samples containing the selected molecules (DBA, T, and M), as depicted in Figure 2, were obtained by dispersing the self-healing filler into the functionalized precursor ECC-R-160 using ultrasonication for 30 min at room temperature. Subsequently, the addition of the hardener and the curing cycle were conducted following the same procedure described earlier. The resulting samples were labeled as EP-R-160-DBA, EP-R-160-T, and EP-R-160-M. FTIR analysis, used to study the functionalization of the epoxy precursor and to estimate the amount of the elastomeric phase bonded to the epoxy precursor for the temperature of functionalization of 160 °C, has been discussed in depth through Figures S1–S3 of the Supplementary Electronic Materials (S.E.M.).

### 2.3. Morphological Analysis by Scanning Electron Microscope (SEM)

Scanning electron microscope (SEM) micrographs to analyze the morphology in terms of dimensions and dispersal state of the rubber domains in the hosting epoxy resin were captured using a field emission SEM apparatus (JSM-6700F, Jeol, Akishima, Japan) operating at 3 kV. Sections of the composites were obtained by cutting solid samples using a sledge microtome. These sections were etched and then gold-sputtered before SEM observation by following a procedure already detailed in Spinelli et al. [38] and in Guadagno et al. [39].

### 2.4. Nanoindentation Tests

**Figure 3:**
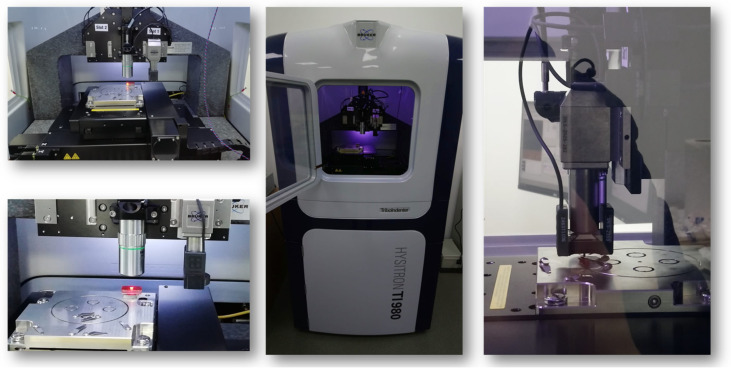
Nanomechanical test system for innovative material characterization.

To evaluate the mechanical properties, nanoindentation tests, accelerated property mapping (XPM), and nanoscratch assessments were conducted using (see Figure 3) a Hysitron TI 980 instrument (Bruker, Billerica, MA, USA) by using a 2D transducer assembly (both normal and lateral force) equipped with a Berkovich probe.

A standard Berkovich indenter typically has a radius of curvature of approximately 150 nm. Tip-area calibration was performed using a standard fused quartz specimen with a notorious elastic modulus (69.6 GPa).

A load control mode was selected for the nanoindentation test, which was conducted by applying a trapezoidal load function with a peak force of 8000 μN (refer to the Results section for details about slopes and durations), whereas nanoscratch experiments involved the application of a constant load scratch function with a maximum force set at 1500 µN.

**Figure 4:**
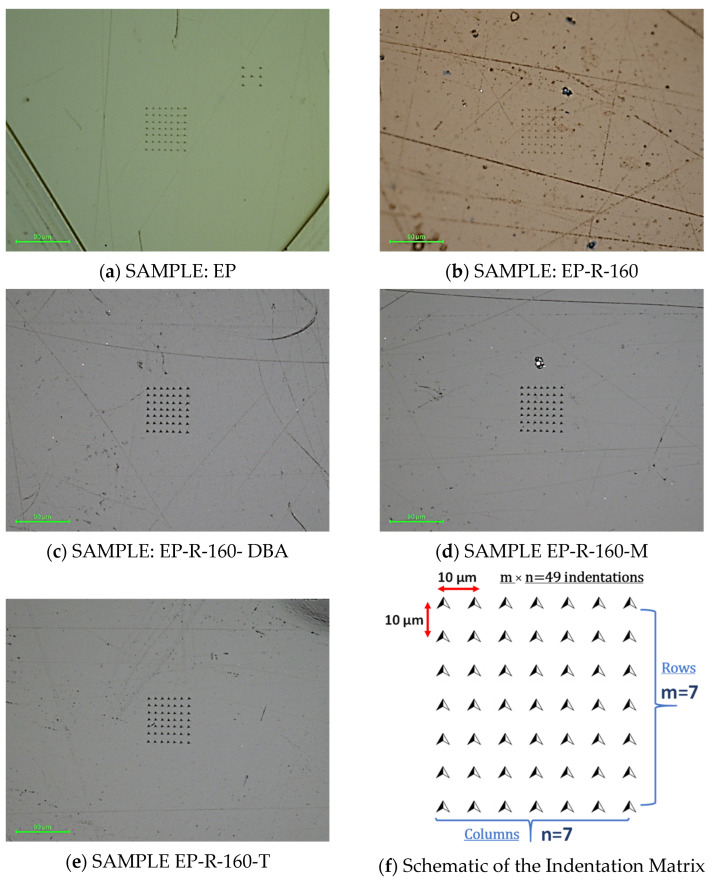
Optical images of XPM nanoindentation test trace made on the surface of (**a**) EP-R, (**b**) EP-R-160, (**c**) EP-R-160-DBA, (**d**) EP-R-160-M, and (**e**) EP-R-160-T. Schematic representation of the indentation matrix in (**f**).

Each test involved 49 indents (arranged in a 7 × 7 grid; indent spacing 10 μm, see Figure 4) to collect statistical data and limit load–displacement curve variability due to not perfectly smooth surfaces of the specimens.

Generally, the XPM testing method follows the same procedure as the standard nanoindentation technique but is expedited by shortening the duration of the functional segments.

In particular, the XPM analysis was performed on a sample surface area measuring 70 × 70 μm, nearing the upper limit of the piezo scanner’s range. Accelerated property mapping was executed utilizing a trapezoidal load function characterized by brief loading, holding, and unloading intervals, each lasting a fraction of a second.

### 2.5. Multiphisic Simulation Study of Nanomechanical Properties

In this study, the nanomechanical behavior in terms of local displacement, hardness, and von Mises stress of some selected composites was examined through numerical analysis based on 3D finite element method (FEM). COMSOL Multiphysics^®^ (version 6.1) served as the simulation environment, in which the experimental Berkovich’s tip used for the experimental nanomechanical characterization was faithfully replicated. It is designed with 3D cad software (FUSION 360, version 2024) according to the general information reported on the manufacturer’s data sheet and then imported into COMSOL. This is because the shape of the indenter is recognized as remarkable parameter conditioning the results and insights of nanoindentation tests [40]. Therefore, the authors’ efforts were concentrated on overcoming the limitations of other tip models commonly used for indentation modeling in the literature, which are typically based on sphero-conical or triangular geometries [41,42,43].

It is worth noting that these last aspects have been recently addressed in the literature, highlighting the crucial role of the morphological and structural features of the nanoindentation tip for an accurate investigation [44,45].

**Figure 5:**
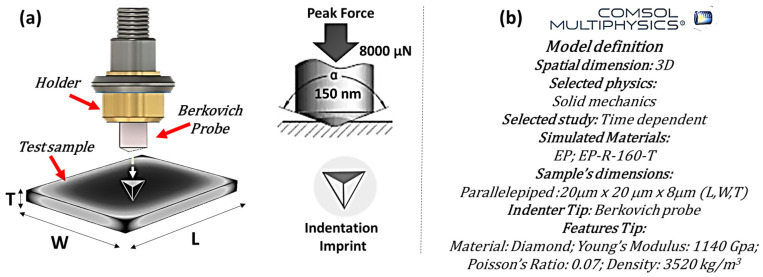
(**a**) Graphic illustration of the simulated case study. (**b**) Crucial model definitions selected for the numerical analysis.

Figure 5a,b provide a schematic depiction of the considered case study and the essential model definitions selected for the simulations, respectively.

**Figure 6:**
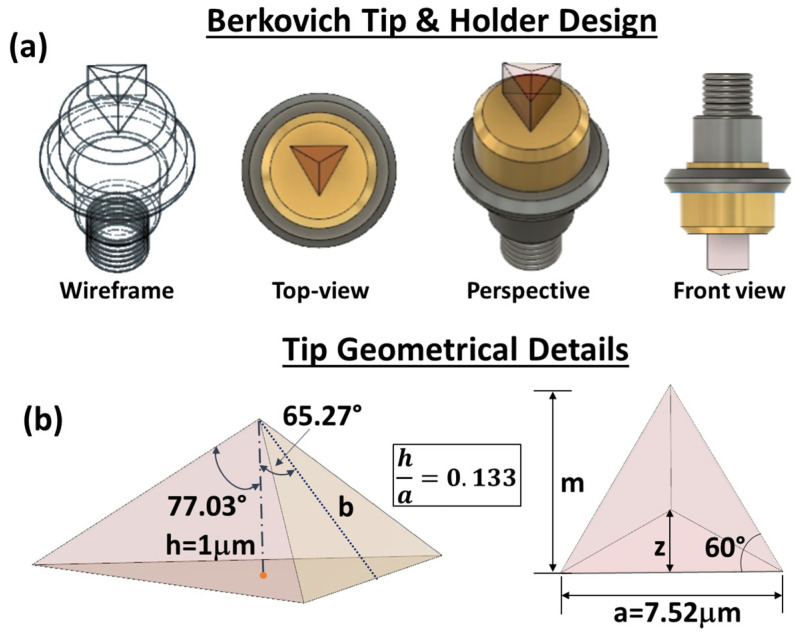
Cad images in (**a**) and geometrical details of the Berkovich’s indenter tip in (**b**).

Given the importance and the role that it plays in simulation results, further details about the indenter tip are reported in Figure 6, which shows some different views of the Berkovich probe in (a) and its geometrical feature in (b).

With reference to the geometrical details shown in Figure 6b, below are the straightforward mathematical steps necessary to determine the projected contact area (hereafter, *A_proj_*) as a function of the indentation depth (*h_i_* = *h·c* with c real coefficient varying in the range [0, ≃1])) between the material and the tip, i.e., *A_proj_* = *f*(*h_i_*).

By considering the following relationships,

(1) $$tan60{}^{\circ}=\frac{m}{a/2};\;m=\frac{\sqrt{3}}{2}a;cos\;65.27{}^{\circ}=\frac{h_{i}}{b}$$

the projected contact area *A_proj_* can be calculated according to the following expression:

(2) $$A_{proj}=\frac{a\cdot m}{2}=\frac{\sqrt{3}}{4}a^{2}.$$

Taking into account the relationship

(3) $$h_{i}=\frac{a\cdot \cos65.27{}^{\circ}}{2\sqrt{3}\sin65.27{}^{\circ}}=\frac{a}{2\sqrt{3}tan65.27{}^{\circ}}$$

the projected contact area *A_proj_* assumes the final expression, as a function of the indentation depth (*h_i_*), in the following form:

(4) $$A_{proj}=3\sqrt{3}h_{i}^{2}{tan}^{2}65.27{}^{\circ}≅24.56\cdot h_{i}^{2}$$

This parameter is crucial for numerically estimating the material’s hardness (*H*). In fact, the hardness is determined from the maximum load (*L_max_*), divided by the projected contact surface, according to the Doerner–Nix model expressed by the following equation [46]:

(5) $$H=\frac{L_{max}}{A_{proj}}$$

## 3. Results

The mechanical characteristics of a material rank among the foremost considerations when designing a structural component. Nanoindentation is a widely recognized technique for assessing mechanical properties at the nanoscale, achieved by tracking displacements of a calibrated indenter tip into a specimen’s surface while applying forces. The resultant force–displacement curve facilitates the derivation of material properties such as elastic modulus and hardness, employing methodologies like the Oliver and Pharr method [47], as well as other interesting mechanical properties investigated in the next subsections, which are strictly correlated to the morphological analysis conducted preventively.

### 3.1. Morphological Investigation

**Figure 7:**
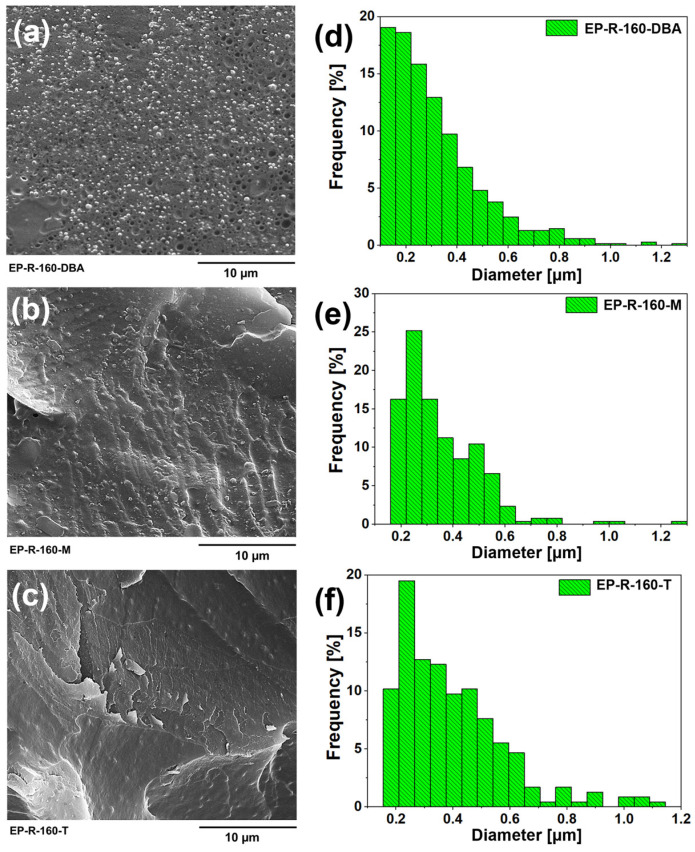
SEM images of the samples EP-R-160-DBA (**a**), EP-R-160-M (**b**), and EP-R-160-T (**c**); rubber domains diameter distribution of EP-R-160-DBA (**d**), EP-R-160-M (**e**), and EP-R-160-T (**f**).

Figure 7 shows the SEM images of the samples EP-R-160-DBA (Figure 7a), EP-R-160-M (Figure 7b), and EP-R-160-T (Figure 7c). All three images show a rubber phase in the form of small globular domains well anchored to the hosting matrix as proof of a successful functionalization. The globular domains of the elastomeric phase are characterized by roughly the same dimensions for all samples. The average globular domains’ diameter ranges between 314 nm (for the EP-R-160-DBA sample), 354 nm (for the EP-R-160-M sample), and 397 nm (for the EP-R-160-T sample). As shown in Figure 7d–f, a monomodal distribution is detected for all samples. Rubber domain diameter distribution was calculated according to a procedure already described in a previous paper [48]. The statistical investigation involved the analysis of over 200 elastomeric domains.

### 3.2. Quasi-Static Nanoindentation

In an initial experimental investigation aimed at the surface characterization of nanocomposite materials, a nanoscale indentation following the Oliver–Pharr method [47] is adopted to evaluate the hardness (*H*) and reduced elastic modulus (*E_r_*) of the samples as well as the respective contact depth and the coefficient of friction (COF) at scratch. In particular, the nanoindentation hardness is determined by dividing the peak load (*P_max_*) by the contact area of the indenter (*A*), whereas the process for calculating the reduced modulus of elasticity entails fitting the unloading section of the load–displacement curve with a power-law function to ascertain stiffness. Subsequently, Er is estimated by establishing the mathematical relationship between unloading stiffness and the projected contact area under load. Notably, this calculation considers elastic deformations in the specimen and the indenter. Indeed, reduced modulus (*E_r_*) in the nanoindentation test is determined using the Sneddon’s formula [49]:

(6) $$\frac{1}{E_{r}}=\frac{\left(1-\nu^{2}\right)}{E}+\frac{\left(1-\nu_{i}^{2}\right)}{E_{i}}$$

where *E* and *ν* represent the elasticity modulus and Poisson’s ratio of the test specimen, while *E_i_* and *ν_i_* denote the elasticity modulus and Poisson’s ratio of the indenter tip.

**Figure 8:**
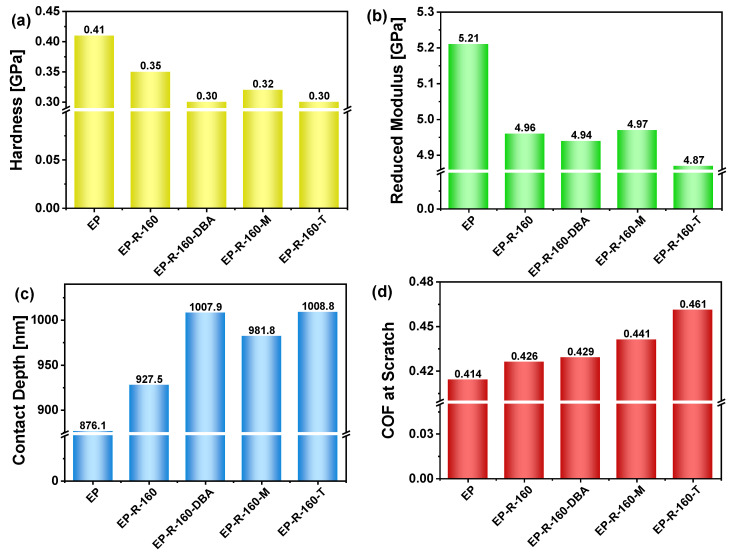
Hardness (**a**), reduced modulus (**b**), and contact depth (**c**) obtained after the XPM nanoindentation at the max force of 8000 μN; coefficient of friction (COF) at scratch (**d**) after nanoscratch at 1500 μN constant load.

The experimental results of the aforementioned mechanical properties are graphically reported in Figure 8a–d, respectively, whereas all numerical results, including statistical information, are summarized in Table S2 of the Supplementary Electronic Materials (S.E.M.).

The findings presented in this study reveal that the hardness of sample EP exhibits an approximately 36.7% increase when compared to samples EP-R-160-DBA and EP-R-160-T (see Figure 8a). The unfunctionalized unfilled epoxy resin (EP sample) tends to be harder than rubber-functionalized epoxy due to its higher crosslinking density and the absence of a rubber phase, which reduces the matrix’s rigidity. The addition of finely well-dispersed globular domains of rubber acting as a plasticizer reduces the overall hardness.

Rubber-functionalized epoxy with self-healing fillers shows a phase composition with nanodispersion domains (as highlighted by the SEM investigation), causing localized deformation and further reducing hardness.

On the other hand, the same observations just reported can justify the results regarding the reduced modulus (see Figure 8b) of the sample EP, which is about 7% higher compared with the other samples. As a consequence of this higher stiffness of the non-functionalized epoxy matrix (EP sample) with respect to the remaining samples analyzed, and as evident from Figure 8c), the contact depth for it is significantly lower (876.1 nm) in comparison with the other measured values. Finally, regarding the coefficient of friction (COF) during scratch testing reported in Figure 8d), sample EP-R-160-T demonstrates the highest coefficient, showing an enhancement of approximately 11.4% and 8.2% compared to sample EP and EP-R-160, respectively.

This enhancement in scratch resistance can be attributed to the incorporation of rubber domains, which alter the material’s mechanical properties and enhance its ability to withstand external forces in lateral displacements.

**Figure 9:**
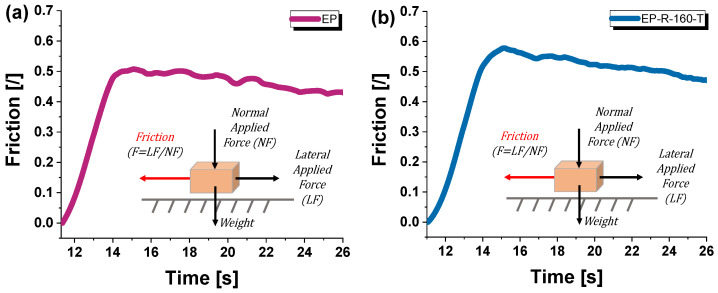
Friction (LF/NF) over time at scratch for (**a**) EP-R and (**b**) EP-R-160-T samples.

In particular, in the rubber-functionalized epoxy, the dispersed rubber phase acts as energy-dissipating regions upon scratching. These rubber domains can absorb and distribute the applied force, reducing the severity of scratches and preventing crack propagation. To complete this analysis, Figure 9 shows the time evolution of the friction during scratching (defined as the ratio between the lateral force LF and normal force NF) for these two mechanically opposing formulations considered in this study, i.e., the unmodified resin (EP) and the EP-R-160-T sample in (a) and (b), respectively.

It is worth noting that, in line with the previously mentioned considerations, the EP-R-160-T sample exhibited greater resistance over time to the strain caused by the indenter scratching movement than the EP sample.

**Figure 10:**
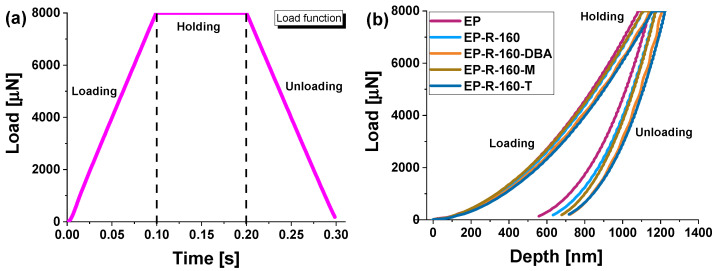
Trapezoidal load function applied for the nanomechanical characterization in (**a**) force vs. displacement curves after accelerated nanomechanical property mapping XPM of all investigated samples in (**b**).

Figure 10 displays average load–displacement curves (from 49 tests), depicting different levels of indenter penetration within the composite samples (see Figure 10b).

Applying a trapezoidal load function with a maximum force of 8000 µN (see Figure 10a) led to a deeper displacement exceeding 700 nm within the material, thereby enabling a more comprehensive assessment of the combined effects on elastic modulus and hardness. The reduced maximum displacement observed in the curves corresponding to the EP sample indicates greater resistance to indenter movement within the material.

This suggests, once again, that this composite has higher mechanical properties, particularly in terms of stiffness, compared to those containing rubber domains (the remaining composites investigated).

**Figure 11:**
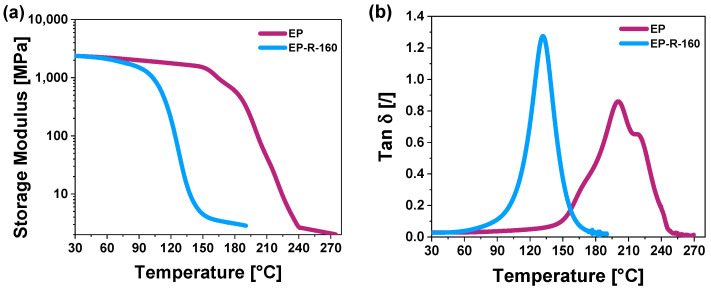
DMA curves for unmodified epoxy sample (EP) and rubber-functionalized specimen (EP-R-160): (**a**) Tan δ vs. temperature; (**b**) storage modulus vs. temperature.

It is interesting to note how the results from nanomechanical characterization align with the experimental findings of Dynamic Mechanical Analysis (DMA) conducted in our previous study [31] and hereafter referenced for clarity and ease of comparison (see Figure 11, which reports the Tan δ and storage modulus vs. temperature for unmodified (EP) and rubber-functionalized (EP-R-160) samples in (a) and (b), respectively).

The sample of unmodified resin (EP), which exhibited higher values of hardness and reduced modulus compared to those functionalized with rubber, showed the highest peak in the mechanical spectrum, related to the glass transition (i.e., Tg), which is centered at 200 °C, whereas for the rubber-functionalized sample (EP-R-160), it appears around 132 °C. Furthermore, it is noticeable that the introduction of rubber also affects the storage modulus. The primary decrease in the storage modulus occurs at lower temperature values for EP-R-160 as a result of incorporating the functionalized precursor into the resin. This is a very relevant result. In fact, a big challenge addressed in the formulation of these self-healing resins was to improve the resin’s dynamic properties and reduce the matrix’s rigidity, acting on its phase composition. Therefore, the design of a material with small domains of the polymer (rubber phase) at higher mobility, finely interpenetrated in the resin, has allowed higher mobility of the chains around the elastomeric domains and a higher efficiency in the auto-repair action. DMA analysis evidences a reduction of the rigidity of the matrix (at the macroscopic level), as evidenced before by the results of “Quasi-static nano-indentation” tests. In Figure 11, it is clearly visible that the peak of Tan δ vs. temperature for the sample containing a rubber phase opens at around 60 °C. This peak is shifted to a lower temperature range with respect to the sample without the rubber phase. It involves a temperature range from 60 °C to 180 °C (with a max around 132 °C), whereas the sample without the rubber phase shows a transition from 140 °C to 250 °C.

### 3.3. Accelerated Property Mapping (XPM) Investigation

**Figure 12:**
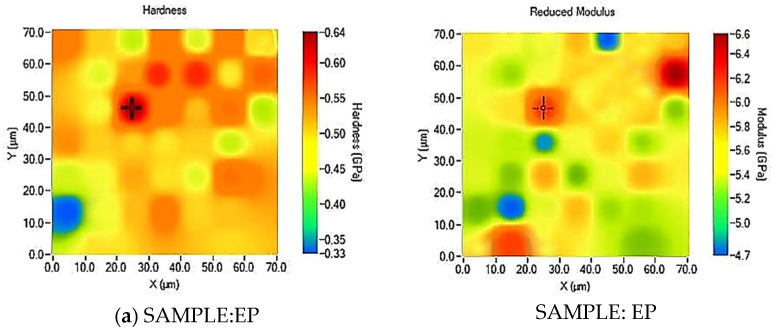

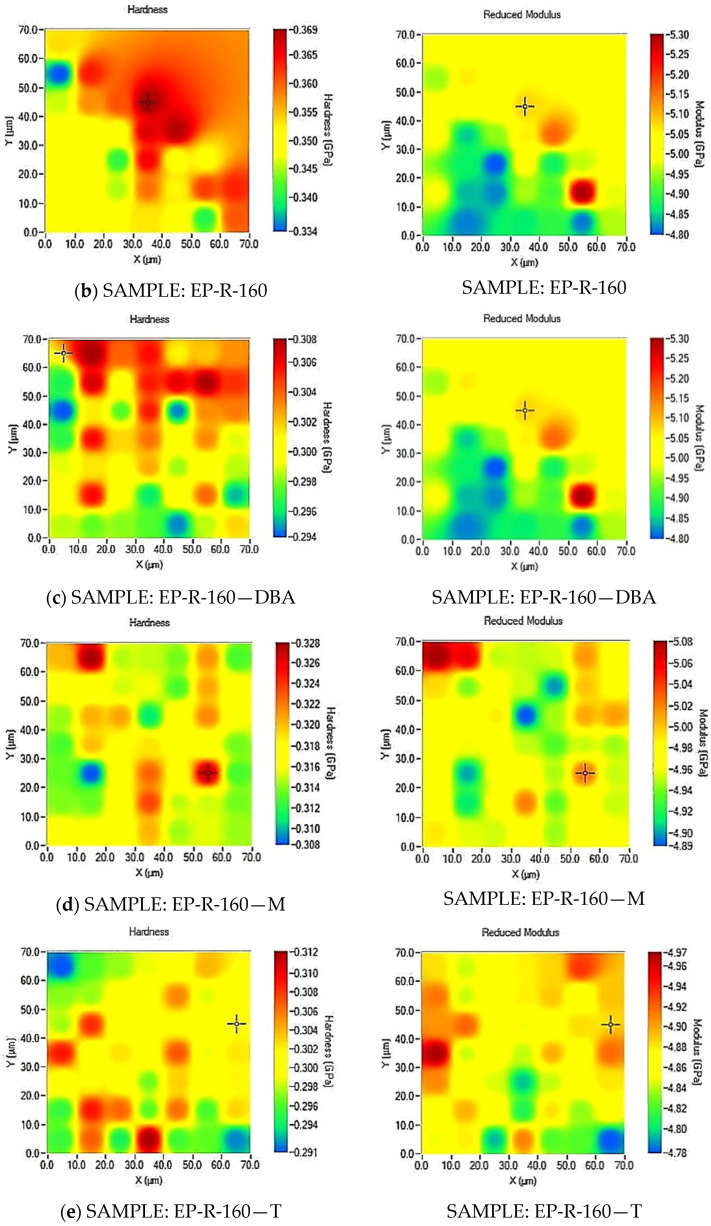
XPM plots of (**a**) EP-R, (**b**) EP-R-160, (**c**) EP-R-160-DBA, (**d**) EP-R-160-M, and (**e**) EP-R-160-T samples.

To understand the mechanical behavior of multiphase materials, it is essential to determine the properties of individual phases and their interactions at the nano- and microscale. Advances in nanoindentation, especially the XPM (rapid indentation) technique, have significantly reduced testing times. XPM, combined with high-resolution microscopy, allows for visualizing hardness and modulus distribution across a sample [50]. It employs a piezo scanner for rapid positioning, creating a grid-like process and generating contour maps of the composite sample. Figure 12 shows the hardness (left parts of the figure) and reduced modulus (right parts) maps determined from the XPM test performed on all formulations considered in the present work. Distinct colors indicate varying levels of the two aforementioned mechanical properties.

Irrespective of the hardness and elastic modulus levels, as indicated by the color bar on the right side of each XPM map, surface mechanical characteristics appear consistent across all samples, particularly for the non-functionalized epoxy resin (see Figure 12a).

This specific composite demonstrates the most homogeneous surface properties compared to the other remaining XPM images, which show, in any case, a good uniformity of surface hardness and reduced modulus, as illustrated in Figure 12b–e.

Concerning the sample EP, the hardness values range from approximately 0.33 GPa (blue) to 0.64 GPa (red). The map shows a relatively uniform distribution, with most areas around 0.45–0.55 GPa (yellow-orange), indicating homogeneity in hardness. For the same sample, the reduced modulus ranges from around 4.7 GPa (blue) to 6.6 GPa (red). The distribution is also fairly uniform, with most regions showing values between 5.0 and 5.8 GPa (green-yellow). The hardness and young modulus values are the highest recorded compared to the other composites analyzed, consistent with the results of the previously illustrated mechanical nanoindentation. With particular reference to samples EP-R-160 and EP-R-160-DBA, the hardness maps show some areas with a sort of variability since some distinct regions of higher (red-orange zone) and lower (green-yellow area) hardness values on their respective surfaces are detected (but in any case, lower than that exhibit by the EP sample). A peak value of 0.369 GPa and 0.308 GPa was detected for the hardness of EP-R-160 and EP-R-160-DBA, respectively. For the remaining composites (EP-R-160-T and EP-R-160-T), there are also minor variations, as medium values of hardness and reduced modulus (green-yellow areas) predominate compared to the respective maximum and minimum recorded values. Overall, the XPM analysis indicates that rubber functionalization and self-healing agents do not introduce remarkable degrees of heterogeneity in hardness and reduced modulus.

These results further validate the authors’ meticulous selection of the chemical composition outlined in the Materials section to prevent the formation of big domains of the elastomeric phase within the resin, thus mitigating observable local inhomogeneity. An overview of the hardness and reduced elastic modulus data obtained from XPM testing suggests minimal contrast compared to values derived from quasi-static nanoindentation. This lack of contrast may be attributed to the average roughness level of the composite surfaces, which hinders the acquisition of highly consistent data. In situ scanning probe microscopy (SPM) imaging of the nanoscratch surface area of the samples, reported in the next subsection, confirms this hypothesis. In other words, the depiction of superficial mechanical features of the nanocomposite sample also serves as an evaluation of its surface homogeneity concerning the nanomechanical behavior revealed by XPM investigation.

**Figure 13:**
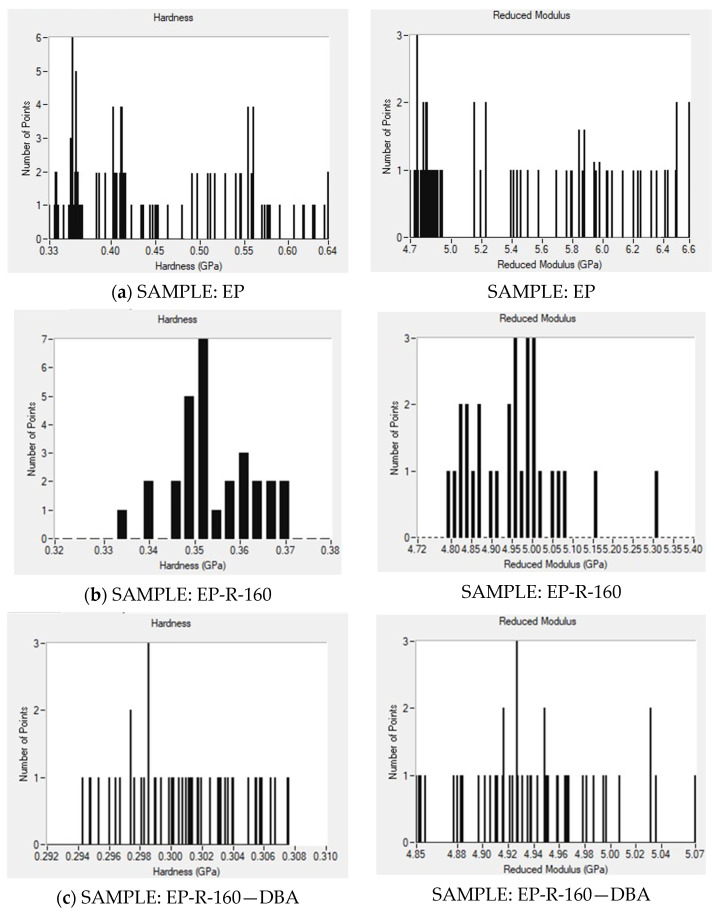

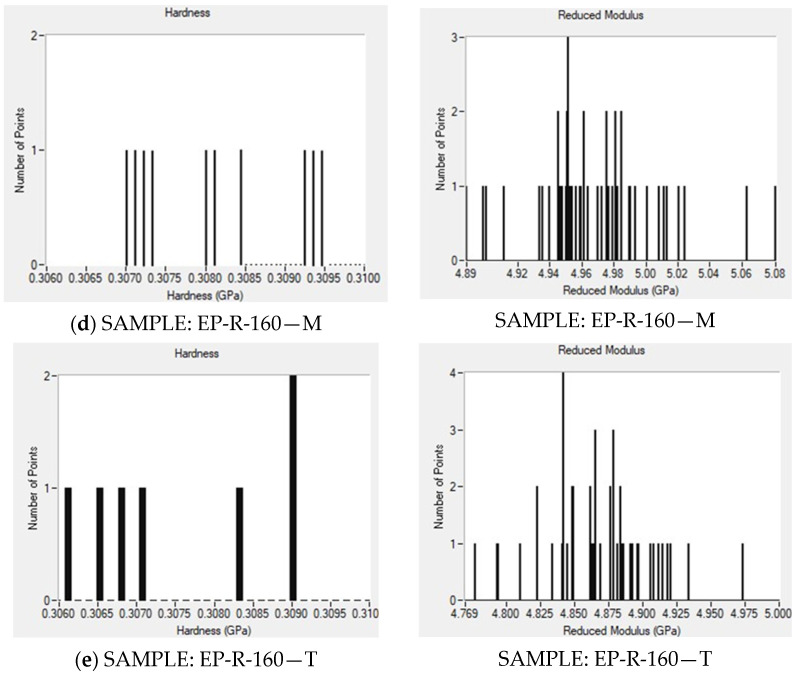
XPM histogram plots of (**a**) EP, (**b**) EP-R, (**c**) EP-R-160-DBA, (**d**) EP-R-160-M, and (**e**) EP-R-160-T samples.

The histogram distribution of nanomechanical parameters for each XPM plot is shown in Figure 13. A weak dispersion of the lines corresponding to hardness and reduced elastic modulus is observed for all investigated samples. This observation is consistent with the nanomechanical XPM plots illustrated in Figure 12. Furthermore, histogram analysis from XPM still shows a good dispersion of rubber domains within the polymer structure. This validates the reliability of the preparation method employed in the present study, considering the tendency of rubber to agglomerate [29], which could result in an imbalanced morphological structure of the composite material.

### 3.4. In Situ Scanning Probe Microscopy (SPM) Inspection

In situ scanning probe microscopy (SPM) integrates nanomechanical testing with high-resolution imaging using a single probe, improving accuracy, repeatability, and efficiency. Unlike ex situ methods, it avoids the need to relocate the sample, thereby saving time and enhancing positioning accuracy.

**Figure 14:**
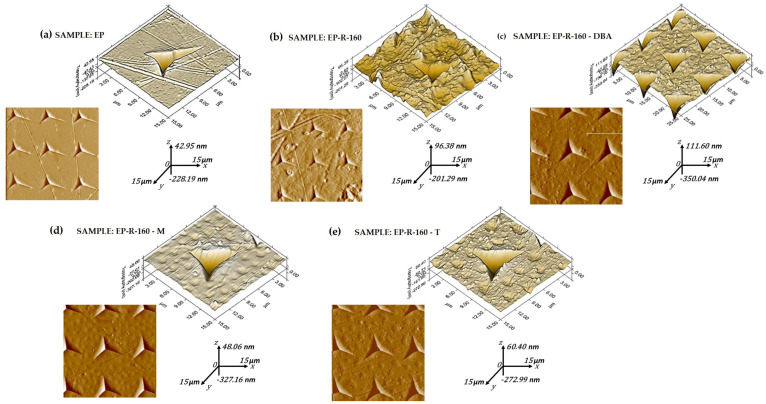
(**a**–**e**) Two-dimensional SPM images of XPM (left parts) and three-dimensional SPM images of XPM (right parts) of nanoindentation test trace made on the surface of (**a**) EP-R, (**b**) EP-R-160, (**c**) EP-R-160-DBA, (**d**) EP-R-160-M, (**e**) EP-R-160-T, and (**e**) samples.

Concerning the foregoing, Figure 14a–e show 2D SPM images (left part) and 3D SPM images (right part) of the XPM nanoindentation test trace made on the surface of all samples taken into consideration in our work.

Observing mainly Figure 14a related to the unmodified epoxy resin (EP sample), it is possible to confirm previous considerations about the surface smoothness and its quality. Rubber-functionalized epoxy resin displays visible imperfections and irregularities and a non-uniform surface finish. These observations support the statements reported in the previous section concerning the XPM analysis, which has shown better uniform surface color maps for the unmodified epoxy compared to the other samples.

**Figure 15:**
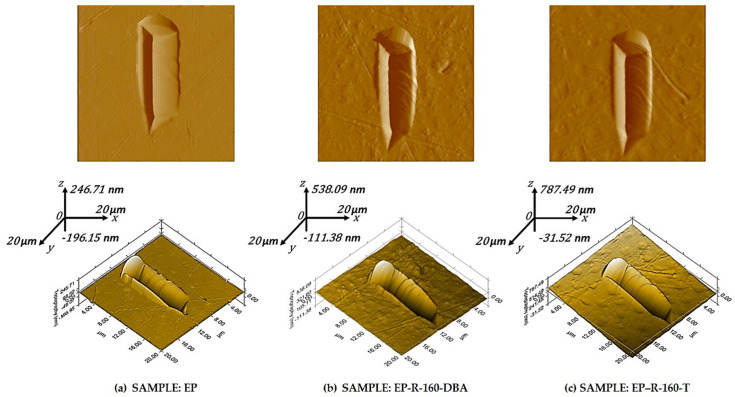
(**a**–**c**) Two-dimensional SPM images (first row) and three-dimensional SPM images (second row) of nanoscratch trace scanned over a 20 μm × 20 μm surface area of (**a**) EP-R, (**b**) EP-R-160-DBA, and (**c**) EP-R-160-T samples.

Another peculiar aspect that provides information on the nanoscratch behavior of composite materials is the trace profile of the groove left by the indenter tip, as depicted in the 2D view (up part) and 3D view (down part) of Figure 15 for the samples EP, EP-R-160 DBA, and EP-R-160—T in (a), (b), and (c), respectively.

The scratch appears noticeably more linear in the case of the unmodified resin (EP sample, see Figure 15a). Conversely, for the other two formulations compared (EP-R-160 DBA, see Figure 15b, and EP-R-160-T, see Figure 15c), the path of the indenter tip appears hindered by the rubbery domains, resulting in jagged and less defined scratch outlines. There is also a perceptible difference in the width of the incision, which can be correlated with the deeper notch made by the probe.

These graphical results agree with the lower scratch resistance of these rubber functionalized-based samples, which was discussed in terms of the coefficient of friction in Section 3.2., Quasi-static nanoindentation.

Furthermore, from the 3D views of Figure 15a–c, it is possible to observe the depth of the tip’s incision, which in the case of the EP sample is lower compared to the other two formulations (EP-R-160-DBA and EP-R-160-T) considered for this comparison via images. This result is consistent with the contact depth values already observed and discussed for these specimens.

### 3.5. Multhyphisics Simulation Study: Results

Finite element (FE) simulation is a well-recognized technique for analyzing the material’s mechanical behavior. Therefore, an experimental–simulation approach is adopted to compare simulation and experimental data. Once the FE model had been validated, it was used to investigate further mechanical properties.

The simulations are conducted focusing exclusively on two materials, EP and EP-R-160-T, which, based on the observed experimental results, are the two most mechanically distinct formulations. The numerical analysis begins with a preliminary investigation over the entire time interval in which the load is applied, aiming to identify the most significant time points for further numerical insights.

**Figure 16:**
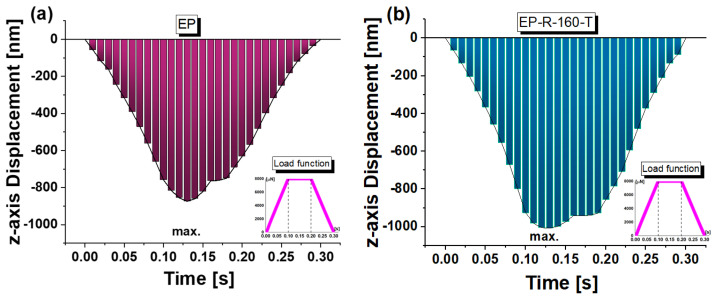
*z*-axis displacement versus the entire time interval for the EP sample in (**a**) and the EP-R-160-T sample in (**b**).

Figure 16 reports the *z*-axis displacement versus the entire time interval (loading, holding, unloading) for the EP sample in (a) and the EP-R-160-T sample in (b). The curves provide detailed insights into the material’s mechanical behavior during the nanoindentation test. At the beginning of this characterization, as the indenter applies force, the penetration depth increases steadily in linear mode. This initial loading stage is pivotal for comprehending the material’s reaction to mechanical strain. Subsequently, there is a holding period during which the load is maintained. This allows for the observation of any viscoelastic behavior or time-dependent deformation of the material. Finally, during the unloading phase, the applied load is removed, and the indenter retreats from the material. The depth rate profile in this phase reveals significant details about the material’s elastic recovery and residual indentation depth.

**Figure 17:**
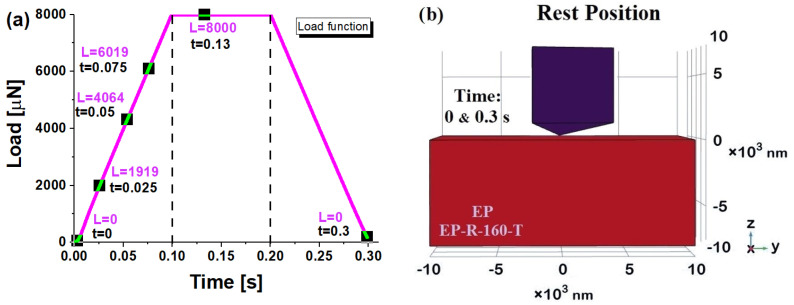
Time instances and corresponding loads selected for particular numerical insights in (**a**); resting position (t = 0 s and t = 0.3 s) with no load (L = 0) for the EP and EP-R-160-T samples in (**b**).

Henceforth, based on the mechanical response over time observed in the previous figure, only some specific time points will be considered as references. More specifically, as indicated in Figure 17a, three instances (0.025 s, 0.05 s, and 0.075 s) were selected during the loading phase, while during the holding interval, the time instance t = 0.13 s was considered, at which the maximum indentation depth was recorded. The corresponding load values are explicitly reported in the figure. Obviously, for the time instances without load (t = 0 and t = 0.3 s), as shown in Figure 17b, the samples are at rest with the indenter tip just tangent to their upper surfaces.

**Figure 18:**
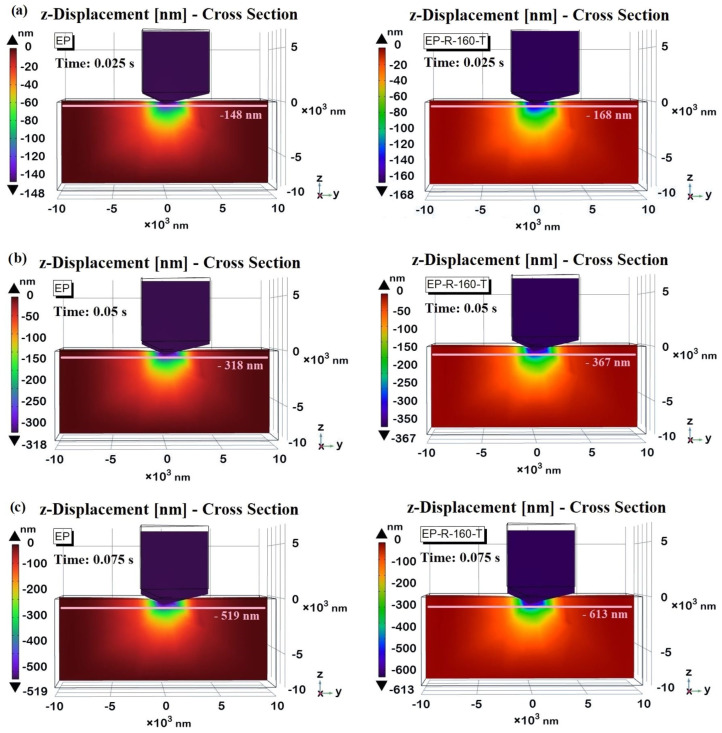
Contact depth for EP sample (left) and EP-R-160-T (right) at some selected time instances during the holding phase: t = 0.025 s in (**a**), t = 0.05 s in (**b**), and t = 0.075 s in (**c**).

Figure 18 reports the simulation findings on the *z*-axis displacement observed for the EP (left part) and EP-R-160-T (right part) for the three-time instances selected during the holding phase, i.e., 0.025 s, 0.5 s, and 0.075 s in (a), (b), and (c), respectively. These views allow us to highlight that, consistent with the results of the previous figure and regardless of the sample, the indentation tip, or equivalently the contact depth, progressively increases over time. In particular, this depth is greater (please refer to the color bar or to the quote lines drawn as guidelines for the eyes) for the EP-R-160-T sample due to its lower hardness and reduced modulus, which have experimentally determined and already been discussed as results.

**Figure 19:**
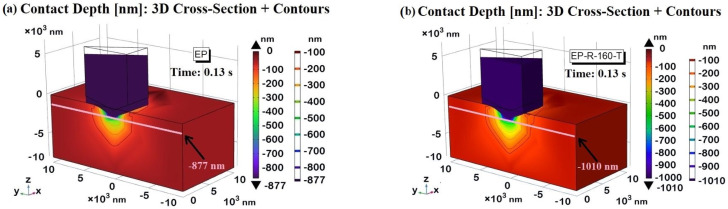
Contact depth for EP sample (**a**) and EP-R-160-T (**b**) at time instances t = 0.13 s, at which the maximum contact depth is recorded. The contour lines highlight the different indentation profiles.

The time instant t = 0.13 s is considered in Figure 19a,b for the EP and EP-R-160-T samples, respectively. At this instant, the maximum contact depth is recorded, measuring −877 nm and −1010 nm for the two samples, which are close to the experimentally obtained results. This particular cross-sectional view allows for a better exploration of the nanoindentation profile. Specifically, the contour lines highlight the greater depth for the EP-R-160-T sample compared to the EP reference according to their hardness values. For the unmodified resin (EP), the z-displacement during nanoindentation is consistently lower. This is because it exhibits higher hardness and stiffness. As the indenter penetrates the surface, the material resists deformation, resulting in a smaller indentation depth. This behavior indicates that the material can withstand higher loads without undergoing significant plastic deformation. In contrast, the sample EP-R-160-T tends to exhibit a greater z-displacement under the same indentation load, reflecting its softer nature. This increased z-displacement is fully compatible with the lower hardness compared to the reference EP. These differences are essential for tailoring materials to specific applications where a balance between hardness and toughness is required.

**Figure 20:**
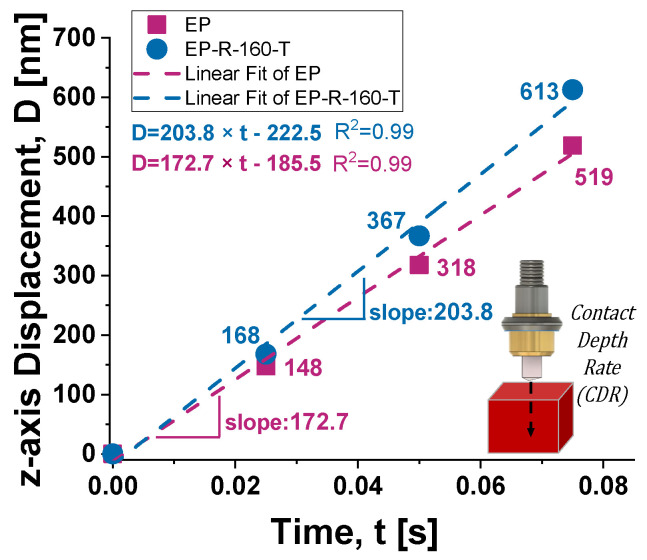
*z*-axis displacement over time during the loading phase to evaluate the depth rate of the samples.

The *z*-axis displacement (D) versus time (depth rate) describes the change in penetration depth of the indenter over time. Once again, this parameter is related to the material’s mechanical properties and, in particular, to its hardness. Figure 20 reports the results concerning the depth rate of the two considered samples evaluated as the slope of the fitting curve (linear fit, R^2^ strictly close to 1) of the displacements of the indenter recording during the loading phase. The EP sample, characterized by a higher hardness compared to the EP-R-10-T, exhibits greater resistance to indenter penetration, and therefore, its depth rate is notably lower: the calculated slopes result in 172.7 and 203.8, respectively, for the two samples.

**Figure 21:**
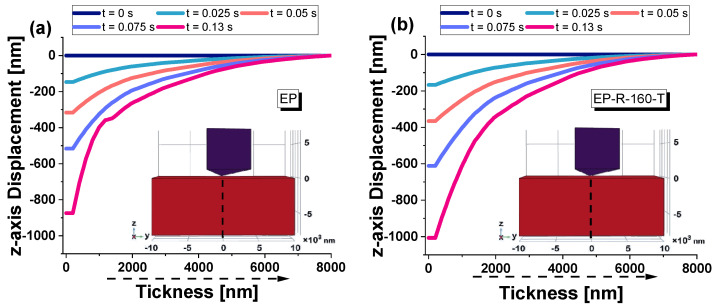
*z*-axis displacement versus thickness at some selected instants of time for (**a**) EP-R and (**b**) EP-R-160-T samples.

In a nanomechanical test of a composite material, the *z*-axis displacement versus thickness can provide information on the deformation behavior of the material under an applied load. In more detail, as the thickness of the material varies, the *z*-axis displacement measures the vertical displacement of the material’s surface in response to the applied force. This relationship is strictly close to the mechanical properties of materials, such as their hardness, elasticity, and resistance to deformation. Figure 21 shows, for the specific time instants considered in this numerical analysis, the *z*-axis displacement vs. the thickness (alongside the segment highlighted in the inset of the same figure) for EP and EP-R-160-T samples in (a) and (b), respectively.

As expected, EP, being a stiffer material, exhibits less displacement for a given applied load, resulting in a smaller *z*-axis displacement versus thickness curve. Conversely, the EP-R-160-T exhibits greater displacement, resulting in a larger curve since it is a softer material.

**Figure 22:**
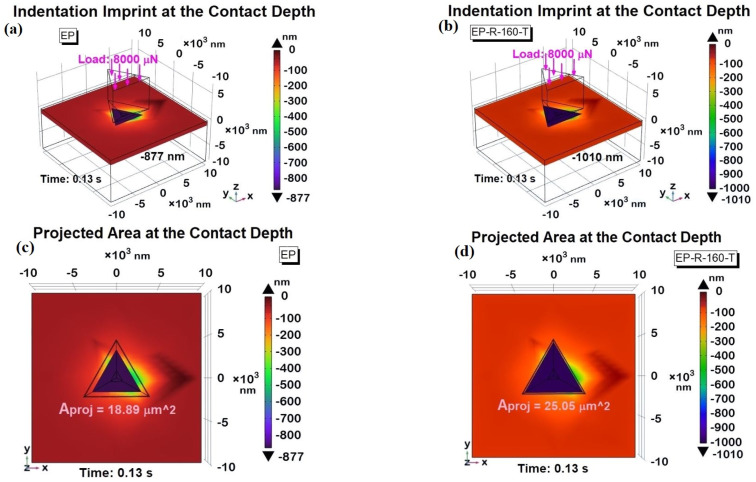
Indentation imprint at maximum contact depth for assessing projected areas (*A_proj_*). A 3D view in (**a**,**b**) for EP and EP-R-160-T sample and corresponding 2D top view in 2D view in (**c**,**d**).

Figure 22 shows a 3D view of the indentation imprint at the maximum contact depth (*h_i_*), i.e., 877 nm and 1010 nm, for the EP sample and the EP-R-160-T sample in (a) and (b), respectively, whereas the corresponding 2D top views are shown in Figure 22c,d. As expected and evident from the analysis of the figures, the projected areas are different according to the relationship *A_proj_* = *f*(*h_i_*). With the greater contact depth reached by the tip in the EP-R-160-T sample, its area is significantly larger than that of the EP sample.

Using Equation (4), the numerically estimated area values are *A_proj_* (EP) = 18.89 µm^2^ and *A_proj_* (EP-R-160-T) = 25.05 µm^2^, respectively.

Once these values were determined and with the maximum applied load (8000 μN) known, the hardness (*H*) of the materials was calculated using Equation (5). The resulting values were 0.42 GPa for EP and 0.32 GPa for EP-R-160-T, closely matching the experimentally obtained values. These numerical results are summarized and compared with the experimental data in Table 1, showing the percentage variation between the two data sets.

**Figure 23:**
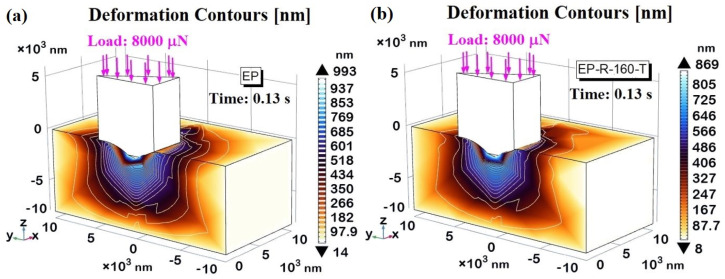
(**a**,**b**) Deformation contours for EP-R and EP-R-160-T samples in (**a**) and (**b**), respectively.

In the nanoindentation investigation, when comparing different composite materials, the displacement magnitude provides further insights into their mechanical properties due to their hardness, elasticity, plasticity, and contact depth differences. Figure 23 shows the deformation contours, detected at the instant t = 0.13 s corresponding to the maximum load of 8000 μN, for the EP sample in (a) and EP-R-160-T in (b). The three-dimensional cross-sectional views are chosen to provide more detail of the resulting indentation profile.

Regardless of the sample, the deformations are highly localized at the contact area between the material/indenter tip interface and along its periphery. It progressively extends into the bulk of the samples, primarily along the *z*-axis direction, where the load is applied. The maximum values recorded in both cases (993 nm for EP and 869 nm for EPR-160-T, respectively) are consistent with the previously presented and discussed contact depth data and hardness values. For the EP sample, being more rigid and thus more prone to crack propagation, it is evident that the deformations extend more significantly in other spatial directions compared to the EP-R-160-T sample. The latter, being relatively softer, better absorbs and dissipates the mechanical energy, reducing the extent of deformations.

Once the model is validated with experimental data and the reliability of the results is confirmed, it can be used to investigate additional mechanical properties that have not yet been here observed experimentally.

For example, in this study, the von Mises stress is numerically investigated since it is a critical measure in engineering and materials science, particularly for predicting when a material will yield under complex loading conditions. It is especially relevant for composite materials, which combine two or more distinct phases to achieve superior mechanical properties compared to the individual components.

**Figure 24:**
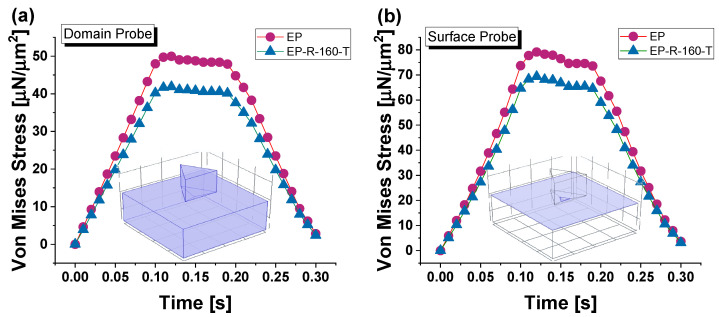
Von Mises stress (average value) for EP-R and EP-R-160-T samples evaluated on the entire domain in (**a**) and upper surfaces of the samples in (**b**), respectively.

Figure 24 depicts the variation of von Mises stress (average values) across the total time span of force application (loading, holding, and unloading phases) for both composites, EP and EP-R-160-T, analyzed over the entire domain and on their upper surfaces (see the inserts of the same figure), in (a) and (b), respectively.

From the analysis of these results, it is evident that, regardless of the exploration area, whether volumetric domain or surface, the von Mises stress remains higher for the EP sample than the EP-R-160-T sample. The maximum stress peak for both samples is reached at t = 0.13 s, corresponding to the maximum penetration depth. Therefore, this particular time instant will be considered for the next investigations. These maximum stress peaks are 49.9 [µN/µm^2^] and 41.8 [µN/µm^2^] over the domain for EP and EP-R-160-T, respectively, and 79.1 [µN/µm^2^] and 69.3 [µN/µm^2^] on their upper surfaces.

**Figure 25:**
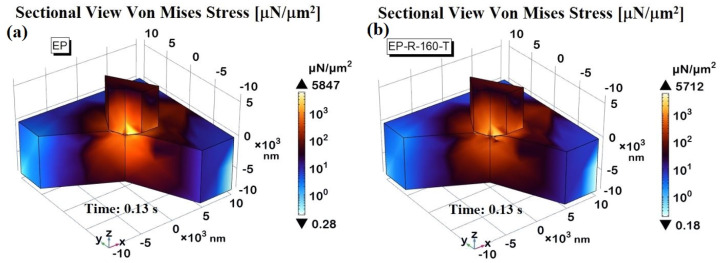
Three-dimensional sectional view of von Mises stress recorded at the time instant t = 0.13 s for EP-R and EP-R-160-T in (**a**) and (**b**), respectively.

Figure 25a,b, respectively, present a 3D view of the two samples properly sectioned to better visualize the spatial distribution of von Mises stress.

The pictures highlight that the main stress values are highly localized at the interface indenter probe/contact surface and near its periphery to extend into the surrounding space progressively. In accordance with the results and considerations already reported in the previous Figure 24a, the higher stress intensity for the EP sample is visually noticeable compared to that of the EP-R-160-T.

**Figure 26:**
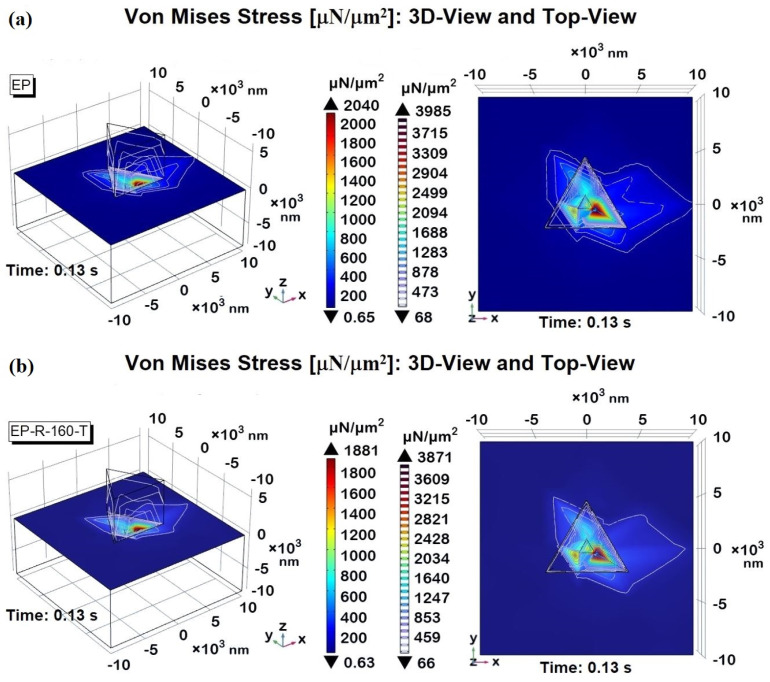
Von Mises stress recorded at the time instant t = 0.13 s for EP-R and EP-R-160-T in a 3D view and top view in (**a**) and (**b**), respectively.

Finally, the von Mises stress profiles detected on the upper surfaces of the materials are further analyzed graphically, with 3D views and top views, in Figure 26a,b correspondingly for the EP and EP-R-160-T samples. These additional graphical representations again highlight and quantify (see the respective color bars) how the higher von Mises stress is concentrated in the indentation area.

The traced contour lines provide information on the von Mises stress distribution on the material surfaces: the appreciable reduced extent exhibited by the EP-R-160-T sample confirms its facilitated energy dissipation and improved ability to absorb impacts, contributing to the overall toughness and resistance to mechanical stresses.

## 4. Discussion

Nanomechanical testing is currently recognized as a valuable technique used to characterize the mechanical behavior of materials at the nanoscale. In particular, when applied to composite materials, it provides crucial insights into their mechanical properties such as integrity, strength, stiffness, toughness, etc. The results serve as valuable feedback for optimizing composite material formulations and processing techniques. Among the various approaches for nanomechanical characterization, experimental nanoindentation and nanoscratch testing are performed in the present study. More specifically, nanoindentation tests are widely indicated to measure the hardness, reduced modulus, and contact depth of materials at the nanoscale. In the field of composites, it helps assess the mechanical properties of the hosting matrix and the influence of any functionalizing agents or dispersed phases on it. By performing nanoindentation tests across different regions of the composite, researchers can evaluate the homogeneity of the material. Additionally, nanoscratch testing investigates materials’ adhesion and wear resistance at the nanoscale. This technique can assess the interface strength between the matrix and its constituents for composite materials.

In our study, the reported result concerning the higher hardness of sample EP (approximately 36.7% more) compared to the values measured for rubber-functionalized epoxy samples EP-R-160-DBA and EP-R-160-T) can be explained on the basis of several factors. First of all, it must be considered that pure epoxy resins usually have a higher crosslinking density compared to rubber-functionalized epoxy. Crosslinking density refers to the number of chemical bonds connecting polymer chains within the material. Higher crosslinking density leads to a more tightly bound network structure, increasing hardness. This rigidity contributes to the hardness of the material. The addition of rubber to the epoxy matrix introduces flexible segments within the material, which can disrupt the uniformity of the crosslinked network. The rubber phase tends to act as a plasticizer, reducing the overall rigidity and hardness of the composite material. Moreover, rubber-functionalized epoxy typically exhibits a more dispersed microstructure, with regions of rubber dispersed within the epoxy matrix. These dispersed rubber domains can act as regions of localized deformation under load, reducing the overall hardness of the material, as also evidenced by DMA investigation.

The same considerations just mentioned justify the result regarding the higher value of reduced modulus for the EP sample (about 7% more) compared to all others investigated and the minor contact depth (only 876.1 nm compared to the 1008.8 measured for the EP-R-160-T sample) associated instead with its resistance to the indentation probe penetration due to the higher hardness value.

In brief, the unmodified resin shows higher hardness and reduced modulus values, while the rubber-functionalized resins display enhanced toughness and energy absorption capabilities due to their limited values of these two mechanical properties.

Conversely, regarding the scratch coefficient of friction, the EP sample exhibits a value of 0.414, which is 11.4% less than the maximum value of 0.461 observed for the EP-R-160-T sample. In rubber-functionalized epoxy, the dispersed rubber phase is relevant in improving the material’s scratch resistance and toughness. When a scratch occurs on the surface, the rubber domains within the epoxy matrix serve as energy-dissipating regions. As a result, when the scratching force is applied, the rubber domains can deform and absorb a considerable amount of the energy, thereby reducing the severity of the scratch. This energy absorption mechanism favors the distribution of the applied force more evenly across the material’s surface, preventing localized damage and minimizing the risk of crack formation and its subsequent propagation. In brief, the dispersed rubber phase in the host epoxy matrix enhances its ability to withstand mechanical stresses, providing superior scratch resistance and toughness compared to the unmodified resin.

For a comprehensive understanding of the mechanical behavior of multiphase materials, such as those examined in this study, it is crucial to ascertain the mechanical properties of individual phases and their mutual interactions at the nano- and microscale. Recent progress in nanoindentation methodologies has notably minimized testing durations, and the XPM (also known as rapid indentation), in conjunction with high-resolution microscopy results, is currently recognized as a valid and fast technique enabling the visualization of hardness and modulus color distribution across a predefined test area of the sample. In brief, XPM expands upon the traditional single indentation approach by employing a piezo scanner to switch positions, transforming it into a grid-like process rapidly. The primary advantage of using the XPM technique lies in its ability to extract valuable insights from the nanoindentation of multiphase polymeric materials, enabling the generation of contour maps for the composite sample. In the present study, this technique was applied to visually assess these two mechanical properties and extract valuable information regarding the surface quality of the prepared samples. Although all composites exhibit fairly acceptable surfaces free of significant defects, confirming the optimal choice of chemical composition and production process found by the authors, an almost uniform and accurate surface is particularly observed for the EP sample.

Our prepared composites do not necessitate further surface finishing or post-processing to achieve the desired surface quality. This result is highlighted by the SPM analysis performed to complement this nanomechanical characterization. Let us point out that in situ scanning probe microscopy (SPM) imaging seamlessly integrates nanomechanical investigation with high-resolution SPM imaging. This technique employs a single probe for both imaging the sample surface and conducting the test, enabling quick on-site image acquisition without relocating the sample. This technical solution enhances testing accuracy, repeatability, and efficiency compared to ex situ imaging methods, which require an additional module like atomic force microscopy (AFM). This translates into increased time required for relocating the sample for imaging and accuracy problems in positioning it on the desired inspection areas.

In a future work, the experimental characterization will be completed with nanodynamic mechanical testing, which can be performed with the same tribo-indenter used and described in the current work.

This study concludes by presenting numerical results obtained through multiphysics simulations conducted with finite element-based software COMSOL Multiphysics^®^. In fact, it is widely recognized simulation studies play a crucial role in understanding the mechanical properties of composite materials, especially at the nanoscale. Conducting experimental tests on composite materials can be time-consuming, labor-intensive, and expensive. Simulation studies offer a cost-effective alternative by allowing researchers to explore various design possibilities and hypotheses before manufacturing the composite and experimental testing.

The first step was to ensure the reliability of the numerical results. They were compared with experimental data for validation. In particular, the experimental/simulated results regarding the contact depth and hardness of two reference composites, EP and EP-R-160-T, were compared by finding that the values are in perfect agreement. These two composites were selected based on the experimental characterization, as they had mechanically less similar properties, and therefore, they were extensively investigated numerically. In addition to the mechanical properties experimentally analyzed, further properties were investigated with the validated simulation model. In the present study, reserving the investigation of further aspects for a future paper, attention has been focused on a fundamental property when discussing mechanical properties: the von Mises stress.

It is often used in engineering and materials science to predict the mechanical behavior of materials under complex loading conditions.

In agreement with the experimental finding, the simulation study confirms that the EP sample with higher hardness and reduced modulus shows less penetration depth under the same applied load. A value of 877 nm (close to the experimental result of 876.1 nm) and 1010 nm (close to the experimental result of 1008.8 nm) were calculated for EP and EP-R-160-T, respectively. The numerical results of the hardness provide a value of 0.42 GPa and 0.32 GPa for EP and EP-R-160-T, respectively, which match the experimental data of 0.41 GPa and 0.30 GPa.

Once again, it is found that the EP sample, given its higher hardness value, is characterized by a more rigid network structure that resists deformation under load, thereby limiting the penetration depth of the indenter. In contrast, the EP-R-160-T sample, due to its compliant behavior, facilitates deeper indentation into the material.

These features strongly affect the von Mises stress profiles, and, as expected from theory, the EP sample exhibits significantly higher stress levels than the other sample taken for comparison (EP-R-160-T). In conclusion, simulation studies are crucial in composite material design by providing predictive capabilities, optimization opportunities, and detailed insights into material behavior. Utilizing computational modeling enables researchers to expedite materials development, minimize costs, and customize composite materials to suit various application needs.

## 5. Conclusions

This paper mainly focused on investigating the nanomechanical behavior of composites based on the unmodified resin (EP sample) and others, which refer to the same resin functionalized with a rubber phase and including molecules able to confer a self-healing ability to the epoxy resins. The goal of this work has been to explore the influence of the rubber domains formed within the resin and the presence of self-healing molecules on the overall mechanical features of the resulting structures. In more detail, using an advanced tribo-indenter apparatus, a nanomechanical characterization was performed in terms of hardness, reduced modulus, contact depth, and coefficient of friction. Additionally, to support the experimental activity, a computational approach was developed with commercial software based on the finite element method (FEM) to deepen the observed results and add knowledge to these advanced materials. Summing up, the main results are listed below:

- Hardness and Modulus: The unmodified resin (EP sample) exhibits a higher hardness (about 36.7% more) and reduced modulus (about 7% more), consequently leading to a lower contact depth and coefficient of friction (COF) (11.4% less) compared to other rubber-functionalized formulations (EP-R-160, EP-R-160-DBA, EP-R-160-M, and EP-R-160-T). In particular, the sample EP-R-160-T demonstrates the highest COF, showing an enhancement of approximately 11.4% and 8.2% compared to sample EP and EP-R-160, respectively. In fact, the addition of finely well-dispersed globular domains of rubber acting as a plasticizer reduces the overall hardness;
- Mechanical Energy Absorption: Other rubber-functionalized formulations (EP-R-160, EP-R-160-DBA, EP-R-160-M, and EP-R-160-T) are better suited for preserving damage from mechanical stresses due to their capabilities in absorbing mechanical energy. In fact, the addition of finely well-dispersed globular rubber domains acting as energy-dissipator upon scratching reduces the severity of scratches and prevents crack propagation;
- FEM Simulation Validation: The excellent agreement between the experimental nanoindentation data and the finite element method (FEM) simulations serves as a strong validation of the model.
  This validation of the FEM model underscores its efficacy in predicting the mechanical behavior of nanocomposite materials under nanoindentation;
- Successful Rubber-functionalization of Epoxy Systems: SEM images of the samples EP-R-160-DBA, EP-R-160-M, and EP-R-160-T, chemically modified with the rubber phase and containing self-healing molecules, show a rubber phase in the form of small globular domains well anchored to the hosting matrix, serving as a proof of a successful functionalization. The globular domains of the elastomeric phase are characterized by roughly the same dimensions for all samples. The average globular domains’ diameter ranges between 314 nm (for the EP-R-160-DBA sample), 354 nm (for the EP-R-160-M sample), and 397 nm (for the EP-R-160-T sample).

These findings highlight the significant impact of the quantity and distribution of rubber domains within the resin on enhancing its mechanical performance and the potential of FEM simulations in aiding the design and optimization of self-healing resins.

## Figures and Tables

**Table 1:** Comparison of experimental/simulation results for the FE model validation.

SAMPLE	Contact Depth Exp.	Contact Depth Simul.	Percent Change	Hardness Exp.	Hardness Simul.	Percent Change
EP	876.1 [nm]	877 [nm]	0.103	0.41 [GPa]	0.42 [GPa]	2.439
EP-R-160-T	1008.8 [nm]	1010 [nm]	0.119	0.30 [GPa]	0.32 [GPa]	6.667

## Data Availability

Data are contained within the article.

## Supplementary Materials

The following supporting information can be downloaded at https://www.mdpi.com/article/10.3390/polym16131849/s1. Table S1. Quantities of each constituent needed to produce 23.58 g of complete mixture of EP-R-160; Table S2. List of the samples and their hardness, reduced modulus, and contact depth obtained after the XPM nanoindentation at the max force of 8000 μN; Coefficient of friction (COF) at scratch after nanoscratch at a constant load of 1500 μN; Figure S1. FTIR spectra of the precursor ECC (black curve), the liquid rubber R (green curve), the blend of ECC-R (red curve), and the blend of ECC-R-160 (blue curve) in the range of 2000–400 cm^−1^; Figure S2. FTIR spectra of the precursor ECC (black curve), the liquid rubber R (green curve), the blend of ECC-R (red curve), and the blend of ECC-R-160 (blue curve) in the range of 4000–800 cm^−1^; Figure S3. FT-IR spectrum of the ECC-R-160 sample; deconvolution relating to the region of the (a) epoxy ring and (b) peak at 1435 cm^−1^ associated with the CH_2_ stretching.
