# Supplementary material for: Nanoindentation Response of Structural Self-Healing Epoxy Resin: A Hybrid Experimental–Simulation Approach
Source: Polymers (Basel). 2024 Jun 28;16(13):1849. doi: 10.3390/polym16131849 (PMC11244422; doi:10.3390/polym16131849)
Supplement: Supplementary file 1 [file polymers-16-01849-s001.zip › polymers-3063271-supplementary.pdf]

Supplementary electronic materials

Nanoindentation Response of Structural Self-Healing Epoxy Resin: A Hybrid Experimental-Simulation Approach.

Giovanni Spinelli [1,2]*, Rosella Guarini [2,3], Evgeni Ivanov [2], Elisa Calabrese [3], Marialuigia Raimondo [3], Raffaele Longo [3], Liberata Guadagno [3]* and Luigi Vertuccio [4]

[1] Faculty of Transport Sciences and Technologies, University of Study "Giustino Fortunato", Via Raffaele Delcogliano 12, 82100 Benevento, Italy

[2] Open Laboratory on Experimental Micro and Nano Mechanics, Institute of Mechanics, Bulgarian Academy of Sciences, Acad. G. Bonchev Str., Block 4, 1113 Sofia, Bulgaria; rgrosagi@gmail.com (R. G.); ivanov_evgeni@yahoo.com (E. I.)

[3] Department of Industrial Engineering, University of Salerno, Via Giovanni Paolo II, 84084 Fisciano, Italy; elicalabrese@unisa.it (E. C.), mraimondo@unisa.it (M. R.), rlongo@unisa.it (R.L.)

[4] Department of Engineering, University of Campania "Luigi Vanvitelli", Via Roma 29, 81031 Aversa, Italy; luigi.vertuccio@unicampania.it

* Correspondence: g.spinelli1@unifortunato.eu (G.S.); lguadagno@unisa.it (L.G.)

Table S1. Quantities of each constituent needed to produce 23.58 g of complete mixture of EP-R-160.

Constituent	EP-R-160 [g]	EP-R-160 [%]
ECC	10.0	42.4
CTBN	1.2	5.1
PPH_3	2.4	10.1
MHHPA	10.0	42.4

Table S2. List of the samples and their Hardness, Reduced Modulus and Contact Depth obtained after the XPM nanoindentation at the max force of 8000 uN; Coefficient of friction (COF) at scratch after nanoscratch at 1500 μN constant load.

**Sample**	**Hardness** [GPa]	**Reduced Modulus** [GPa]	**Contact Depth** [nm]	**COF at Scratch**
EP	**0.41** *(SD: 0.01)*	**5.21** *(SD: 0.03)*	**876.1** *(SD: 0.4)*	Test 1: 0.401<br>Test 2: 0.421<br>Test 3: 0.419<br>***Mean: 0.414***
EP-R-160	**0.35** *(SD: 0.01)*	**4.96** *(SD: 0.02)*	**927.5** *(SD: 0.2)*	Test 1: 0.432<br>Test 2: 0.417<br>Test 3: 0.430<br>***Mean: 0.426***
EP-R-160 - DBA	**0.30** *(SD: 0.00)*	**4.94** *(SD: 0.02)*	**1007.9** *(SD: 0.1)*	Test 1: 0.432<br>Test 2: 0.425<br>Test 3: 0.43<br>***Mean: 0.429***
EP-R-160 - M	**0.32** *(SD: 0.00)*	**4.97** *(SD: 0.01)*	**981.8** *(SD: 0.2)*	Test 1: 0.440<br>Test 2: 0.455<br>Test 3: 0.429<br>***Mean: 0.441***
EP-R-160 – T	**0.30** *(SD: 0.00)*	**4.87** *(SD: 0.02)*	**1008.8** *(SD: 0.1)*	Test 1: 0.455<br>Test 2: 0.463<br>Test 3: 0.466<br>***Mean: 0.461***

SPECTROSCOPIC ANALYSES

The spectroscopic analyses were carried out on the ECC-R-160 liquid mixture to investigate the functionalization reaction's efficiency at the chosen temperature of 160 °C. FTIR spectra of the epoxy precursor ECC, liquid rubber R, and ECC-R blend were compared with the spectrum of the ECC-R-160 blend (see Figures S1 and S2). The liquid blend ECC-R corresponds to the epoxy mixture precursor/elastomer raw before the heat treatments for 15 h. Focusing the attention on Figure S1, in the range between 1650 cm^{-1} and 1850 cm^{-1} (see inset on the left), the spectrum of the rubber phase shows two absorption bands for the ester carbonyl group,

as a consequence of the hydrogen bond interactions established among the molecules of the liquid rubber. In particular, the band at 1738 cm^{-1} is ascribed to the free ester carbonyl group, while the band at 1710 cm^{-1} belongs to the H-bonded carbonyl group, involved in the hydrogen bond interactions with the hydroxyl groups of the same rubber molecules. In the same range of wavenumber, the spectrum of the precursor shows the ester C=O stretching band, around 1730 cm^{-1}, while the spectrum of the ECC-R blend displays broadband always at 1730 cm^{-1}, which belongs to the carbonyl groups of both the components. In the same region of wavenumber, it is possible to observe the presence of a shoulder peak at 1780 cm^{-1} that could be considered the experimental evidence of the functionalization reaction between the oxirane ring of the epoxy precursor and the carboxylic groups of the rubber phase. This effect is due to the presence of an electron-withdrawing group (hydroxyl group in β position) that can determine the shift of the carbonyl signal to higher values of wavenumber for inductive effect. The peak at 1120 cm^{-1} (see inset on the right of Figure S1), assigned to the C-O stretching of the secondary alcohol generated by the opening of the epoxy group during the functionalization reaction, supports the hypothesized mechanism. Further confirmation of the occurred functionalization is deduced by the results depicted in Figure S2, in the range of wavenumber between 3700 cm^{-1} and 3100 cm^{-1}. ECC-R-160 sample shows an absorption band at 3350 cm^{-1} ascribed to the –O.H. groups generated during the reaction. In addition, the absorption band at 3230 cm^{-1}, ascribed to the hydroxyls of the –COOH group of the R elastomer, disappears after the functionalization reaction. As a consequence of the functionalization reactions, the peak at 913 cm^{-1}, ascribed to the oxirane group of epoxy precursor, decreases in intensity (see the right inset of Figure S2).

More in particular, the reduction of the peak relative to the oxirane group was evaluated, normalizing the peak at 913 cm^{-1} to the peak at 1435 cm^{-1} associated with the CH_2 stretching of six terms ring, which is assumed chemically unmodified during the reaction. The ratio ($R = A_{peak\ 913}/A_{peak\ 1435}$) of the subtended areas was evaluated for precursor-liquid rubber system before and after the functionalization process, respectively. An algorithm based on the Levenberg–Marquardt method to separate the individual peaks in the case of unresolved, multicomponent bands, was applied. To reduce the number of adjustable parameters and to ensure the uniqueness of the result, the baseline, the band shape, and the number of components were fixed. The minimum number of components was evaluated by visual in the section based on abrupt changes in the slope of the experimental line shape. The program calculated, by a non-linear curve fitting of data, the height, the full-width half height (FWHH), and the position of the individual components. The peak function was a mixed Gauss–Lorentz line shape of the form, reported in Equation (S1):

$$f(x) = (1-L)H\exp\left[-4\ln(2)\left(\frac{x-x_0}{w}\right)^2\right] + LH\left[4\left(\frac{x-x_0}{w}\right)^2+1\right]^{-1} \quad \text{(S1)}$$

where x_0 = the peak position; H = peak height; w = FWHH; L = fraction of Lorentz character.

The results of this deconvolution procedure for the functionalized precursor at 160°C, in the above-mentioned ranges of wavenumbers, are shown, respectively, in Figure S3 (a,b). This procedure was repeated for the systems rubber-precursor before and after the functionalization process at 160°C.

A reduction of 13.5% was found for ECC-R-160 system. This is further proof that the higher temperature value allows for obtaining a greater amount of bond between the carboxyl group of the rubber and the epoxy ring of the precursor, making the precursor functionalization more efficient. The most effective functionalization process affects the resin structure and consequently the thermal and mechanical properties.

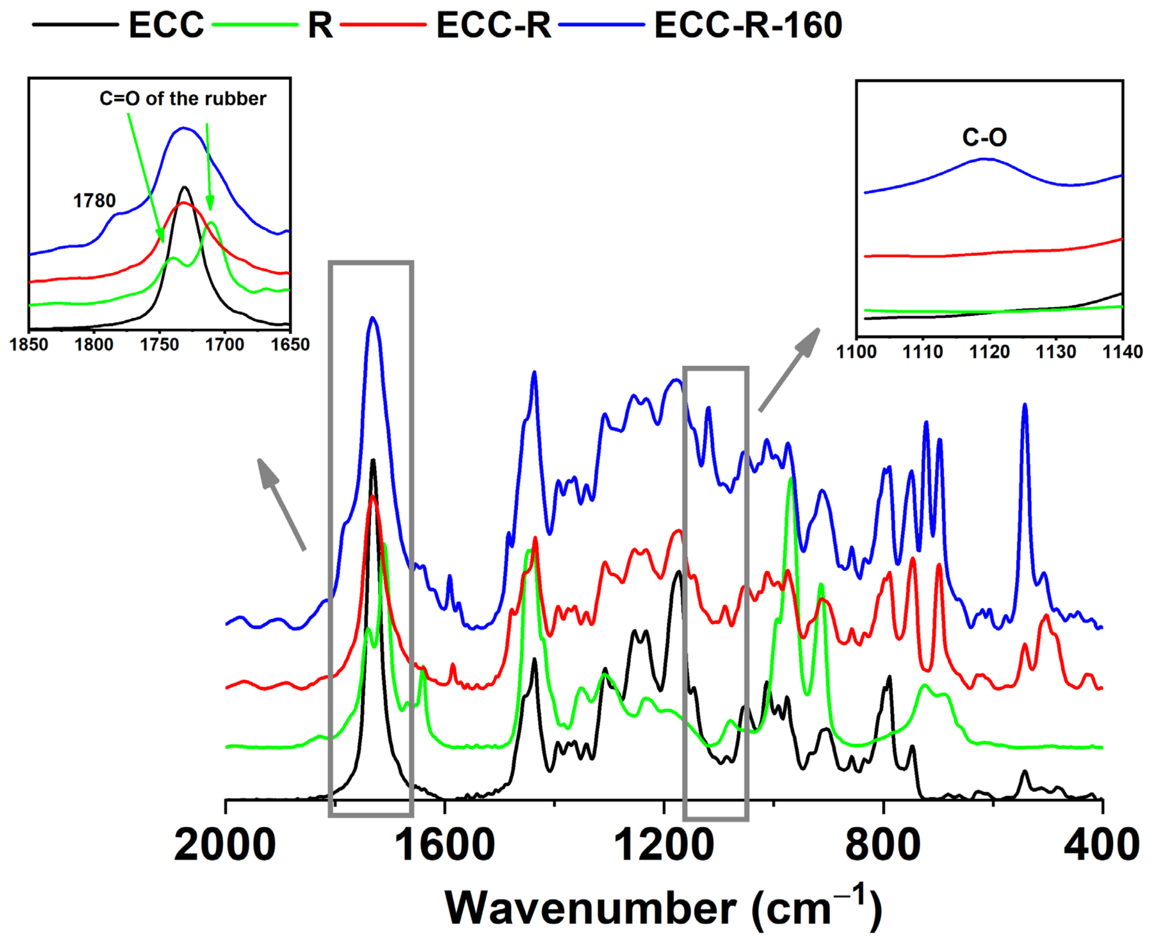

Figure S1. FTIR spectra of the precursor ECC (black curve), the liquid rubber R (green curve), the blend of ECC-R (red curve), and the blend of ECC-R-160 (blue curve), in the range 2000–400 cm^{-1}.

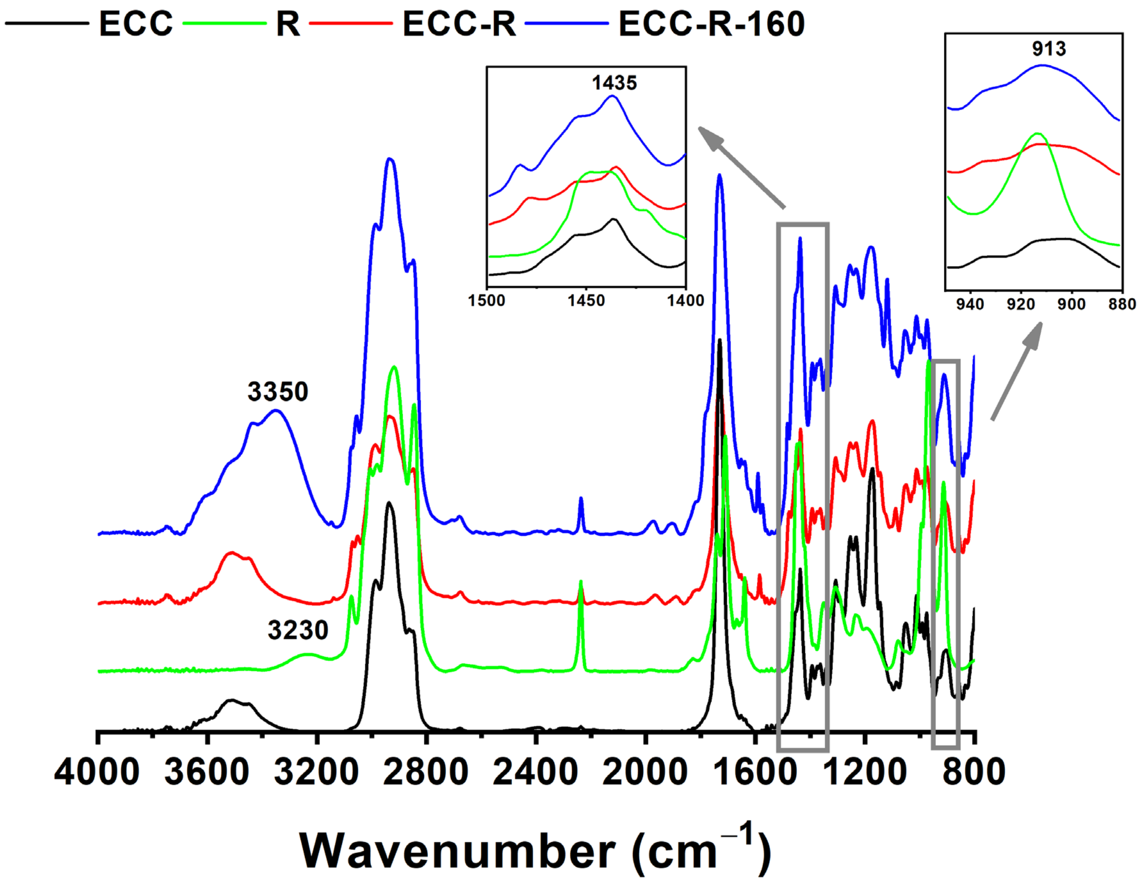

Figure S2. FTIR spectra of the precursor ECC (black curve), the liquid rubber R (green curve), the blend of ECC-R (red curve), and the blend of ECC-R-160 (blue curve), in the range 4000–800 cm^{-1}.

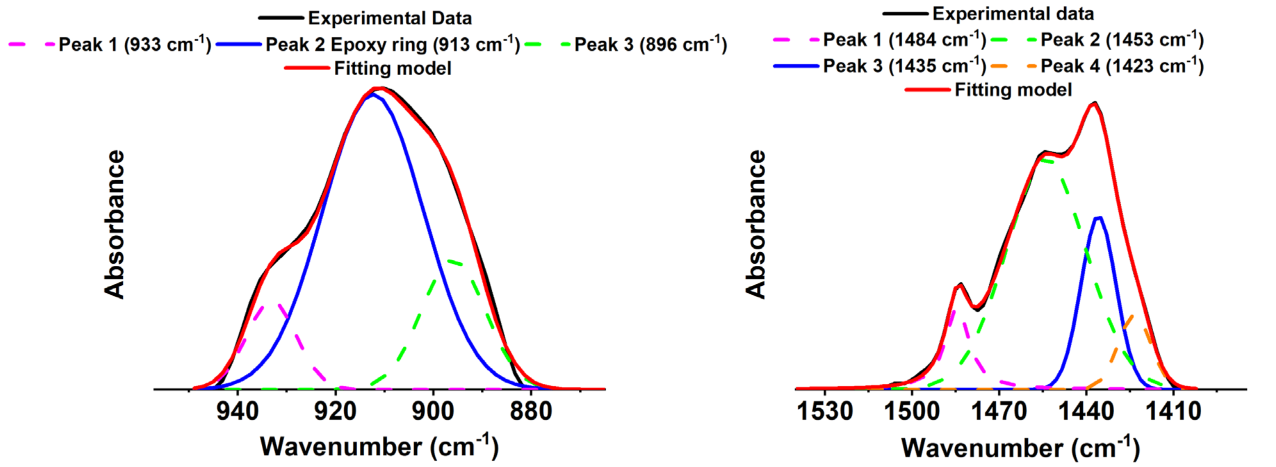

(a) (b)

Figure S3. FT-IR spectrum of the ECC-R-160 sample; deconvolution relating to the region of the (**a**) epoxy ring; (**b**) peak at 1435 cm^{-1} associated with the CH_2 stretching.
